# EEG microstates dynamics of happiness and sadness during music listening

**DOI:** 10.3389/fnhum.2025.1472689

**Published:** 2025-06-18

**Authors:** Ashish Gupta, Chandan Kumar Srivastava, Braj Bhushan, Laxmidhar Behera

**Affiliations:** ^1^Department of Electrical Engineering, Indian Institute of Technology, Kanpur, India; ^2^Department of Humanities and Social Sciences, Indian Institute of Technology, Bombay, India; ^3^Department of Humanities and Social Sciences, Indian Institute of Technology, Kanpur, India; ^4^School of Computing and Electrical Engineering, Indian Institute of Technology, Mandi, India

**Keywords:** EEG microstate, emotion, music, attention, mind-wandering

## Abstract

The human brain naturally responds to music, with happy music enhancing attention and sad music aiding emotion regulation. However, the specific electroencephalogram (EEG) microstates linked to these cognitive and emotional effects remain unclear. This study investigated the microstates associated with happiness and sadness, focusing on the alpha band, using classical music as stimuli. Results revealed a significant increase in class D microstate, associated with attention, during happy music listening. An inverse relationship between class C (linked to mind-wandering) and class D microstates was observed. Analysis of global explained variance (GEV) and global field potential (GFP) indicated that happy music upregulated class D and downregulated class C microstates compared to baseline. In contrast, sad music elicited an increased presence of class B, class C, and class D microstates, with GEV and GFP analyses showing upregulation of class C and class D compared to the resting state. These findings suggest distinct cognitive effects: (1) an increase in class D and reduction in class C microstates explain enhanced attention during happy music listening, and (2) the concurrent upregulation of class C and class D microstates underpins enhanced emotion regulation and self-regulatory goals observed upon sad music listening. Notably, compared to baseline, the mean microstate duration was significantly longer for both happy (*p* = 0.018) and sad (*p* = 0.0003) music, indicating that music listening enhances the temporal stability of active microstates. These findings advance the understanding of the neural mechanisms underpinning music's cognitive and emotional effects, providing a framework to explore music-induced changes in brain dynamics and their implications for emotion regulation and attentional modulation.

## 1 Introduction

Music possesses a unique ability to influence various musical as well as non-music domains, including intelligence (Rauscher et al., 1993; Rideout and Laubach, 1996), attention (Putkinen et al., 2017; Markovic et al., 2017; Jäncke et al., 2015), emotion (Van den Tol et al., 2016), and the Default Mode Network (DMN) (Trost et al., 2012; Wilkins et al., 2014). The DMN is a neural system primarily associated with internally focused cognitive processes, including daydreaming, mind-wandering, self-referential thinking, reminiscing about the past, and planning for the future (Yeshurun et al., 2021). The broad impact of music has led to positive effects on cognitive, motor, emotional, and social functioning in both healthy individuals and those with aging or neurological conditions (Särkämö, 2018). Cognitive functions such as attention and emotion regulation are essential processes for normal human functioning, and music has been used as a supplementary tool to enhance these functions (Rauscher et al., 1993; Rideout and Laubach, 1996; Putkinen et al., 2017; Markovic et al., 2017; Jäncke et al., 2015).

Studies have shown that even brief exposure to music can enhance the spatiotemporal performance (Rauscher et al., 1995; Wilson and Brown, 1997; Rauscher et al., 1993; Rideout and Laubach, 1996) of individuals, often referred to as the Mozart effect. Furthermore, research suggests that musical stimuli (Mammarella et al., 2007) capable of inducing a moderate arousal and pleasant mood in individuals can lead to significant improvements in several cognitive performance (Schellenberg and Hallam, 2005; Schellenberg et al., 2007). Pleasant music stimulates brain regions associated with memory, attention, cognition, and IQ (Verrusio et al., 2015). Recent research (Gupta et al., 2018) suggests that music affects the cognitive system, enhancing brain efficiency through three distinct mechanisms. First, it activates specific regions of the brain in the prefrontal and occipital lobes, which are responsible for IQ and attention. Additionally, music reduces unwanted brain activities, effectively minimizing interference and optimizing cognitive processes.

People generally gravitate toward happy music (Van den Tol et al., 2016) and strive to avoid sadness in their lives. However, paradoxically, they exhibit a strong inclination toward sad music (Taruffi and Koelsch, 2014), particularly during adverse moments' ranging from everyday struggles to relationship difficulties and profound experiences such as the loss of a loved one (Hanser et al., 2016). Research has provided evidence that sad music can evoke a pleasurable experience characterized by a sense of solace (Van den Tol et al., 2016) and profound beauty (Sachs et al., 2015). This feeling is different from real-life sadness (Gupta et al., 2023; Taruffi and Koelsch, 2014; Sachs et al., 2015). The positive effects of listening to sad music on managing difficult circumstances have been extensively studied and well-documented (Van den Tol et al., 2016; Van den Tol and Edwards, 2013; Hanser et al., 2016).

Sad music is frequently sought after by healthy adolescents and young adults as a means of seeking solace (Van den Tol et al., 2016), consolation (Ter Bogt et al., 2017), comfort (Taruffi and Koelsch, 2014), and coping with their emotions (Van den Tol et al., 2016). Listening to sad music during challenging situations consistently serves various self-regulation goals in the cognitive, social, memory retrieval, distraction, mood enhancement, and affect re-experience domains (Van den Tol et al., 2016; Van den Tol and Edwards, 2013). Furthermore, a recent study (Gupta et al., 2023) has shown that listening to sad music after recalling a personal sad event is associated with improved emotion and memory processing, as well as improved alertness. Findings suggest that sad music can have a profound impact on our emotional and cognitive experiences, facilitating the processing and regulation of emotions in challenging situations.

A comparative study (Taruffi et al., 2017) found that happy music had a notable positive impact on meta-awareness, while sad music exhibited a considerable rise in mind-wandering when contrasted with happy music. This is further supported by the heightened centrality observed within the core nodes of the DMN during sad music listening compared to happy music (Taruffi et al., 2017). The DMN has been recognized as the key network associated with mind-wandering (Mason et al., 2007; Kucyi et al., 2013; Andrews-Hanna et al., 2010a,b; Christoff et al., 2009). Numerous other studies have consistently linked the DMN activity to music listening (Trost et al., 2012; Wilkins et al., 2014; Janata, 2009; Ford et al., 2011; Brattico et al., 2011). It should be noted that the experience of wandering of the mind while listening to sad music is different from ordinary wandering of the mind and is characterized by a unique blend of melancholy and pleasure associated with sad music (Gupta et al., 2023; Taruffi and Koelsch, 2014; Sachs et al., 2015) and comprises of spontaneous, self-referential thoughts, emotions, and cognitive processes (Taruffi et al., 2017).

However, the field of music research is confronted with several obstacles, including the lack of a consistent scientific method for delivering musical interventions, the tendency to reduce its effects to surface-level emotional or esthetic experiences, and an incomplete understanding of how the brain functions while engaging with music. To address these issues, detailed and comprehensive studies are essential to reveal the deeper impact of music on cognitive abilities such as attention and emotion regulation. This line of research has the potential to reshape strategies in mental healthcare, educational methodologies, and cognitive therapy, paving the way for innovative and non-intrusive tools to enhance quality of life.

The application of EEG microstates, which represent distinct and non-overlapping topographies (Khanna et al., 2015; Koenig et al., 2002) in recorded electrical signals, has become increasingly popular in the field of electrical neuroimaging. EEG microstates, representing brief instances of coordinated electrical activity in the brain enduring tens of milliseconds, are considered quasi-stable functional states (Michel and Koenig, 2018). One notable advantage of the microstate method is the reliability and comparability of the topographies obtained across different studies (Khanna et al., 2015; Michel and Koenig, 2018), regardless of the number of electrodes used (Zhang et al., 2021), instructions given to participants (such as open or closed eyes) (Zanesco et al., 2021), or the frequency range analyzed (Férat et al., 2022). Importantly, these microstates have demonstrated the potential to function as biomarkers (Schiller et al., 2020) for neuropsychiatric disorders (Soni et al., 2019; Michel and Koenig, 2018), including mood and anxiety disorders, as well as Alzheimer's disease (Al Zoubi et al., 2019; Tait et al., 2020). Recently, it has been applied across a diverse array of studies, encompassing brain resting states (Schiller et al., 2020), neuropsychiatric disorders (Nishida et al., 2013; Soni et al., 2019; Terpou et al., 2022), sleepiness (Cantero et al., 1999), and task-based brain activities (Seitzman et al., 2017; Hu et al., 2023; Gu et al., 2022; Jiang et al., 2024).

Research has consistently identified specific spatiotemporal brain microstates in independent studies, commonly categorized into four distinct classes, A, B, C, and D, based on their unique topological orientations. Map A is characterized by a left-right orientation, Map B by a right-left orientation, Map C by an anterior-posterior orientation, and Map D by a fronto-central maximum. This labeling convention has been widely adopted in various studies (Michel and Koenig, 2018; Hu et al., 2023; Pal et al., 2021; Liu et al., 2021; Pascual-Marqui et al., 2014). Each microstate is associated with specific functions, namely auditory information processing, visual information processing, DMN, and attention (Khanna et al., 2015; Michel and Koenig, 2018; Koenig et al., 2002). A recent review (Tarailis et al., 2023) on the functionality of EEG microstates has additionally associated class A with arousal. The author finds that in addition to visual processing by class B microstate, it plays a key role in scene visualization and self-representation within those scenes (Bréchet et al., 2019). It is frequently observed during tasks involving autobiographical memory (Bréchet et al., 2019). Furthermore, microstate B exhibits a stronger propensity to transition to microstate C (Bréchet et al., 2019), which is linked to the self-experience system. The review further finds that class C relates to mind-wandering specifically to self-reflection and self-referential processes (Bréchet et al., 2019; Custo et al., 2017), while class D is linked to executive functioning, including processes such as working memory and attention (Bréchet et al., 2019; Kim et al., 2021).

Emotional states tend to evolve gradually, whereas EEG signals fluctuate rapidly, leading to significant variability in the features derived from them. Consequently, Chen et al. (2021) propose that examining EEG microstates provides a more nuanced understanding of emotions than conventional EEG analyses. Emotional research has benefited from the successful utilization of microstate analysis (Prete et al., 2022; Chen et al., 2021; Coll et al., 2019), which has the potential to enhance emotion classification (Chen et al., 2021; Shen et al., 2020). The research findings indicate that the four microstates successfully capture the dynamic attributes of emotions (Prete et al., 2022; Hu et al., 2023). However, research investigating the microstates' underpinnings of basic emotions in music (especially audio) is very limited. In addition, to ensure consistency and allow precise neurophysiological interpretations in our current investigation, we chose four microstates that have shown reliability in previous research studies (Prete et al., 2022; Hu et al., 2023; Khanna et al., 2015; Michel and Koenig, 2018; Koenig et al., 2002).

Although microstate topographies are believed to be unrelated to oscillatory activity (Férat et al., 2022) and various approaches (Zulliger et al., 2022), the alpha bands have been identified as the primary driving force behind microstates (Milz et al., 2017). These alpha oscillations can also affect the number of peaks in the global field power (GFP). The periodic nature of EEG microstates is associated with the alpha band rotating phase observed during periods of rest (von Wegner et al., 2021). Multiple studies have demonstrated that the alpha band microstates outperform those of other frequency bands in classifying conditions such as eyes open or eyes closed (Férat et al., 2022), as well as emotions (Shen et al., 2020). A recent EEG microstate study highlighted the efficacy of the alpha band (8–13 Hz) in examining the impact of happy and sad music on the brain (Gupta et al., 2025). Based on this, our investigation focused specifically on the alpha band.

In summary, this study investigates the brain's microstates associated with the fundamental emotions of happiness and sadness within the alpha band. It also seeks to uncover the neural mechanisms underlying the observed cognitive and emotional enhancements during music listening.

As previously discussed, music is known for its ability to influence both emotional states and cognitive functions. Research suggests that listening to happy music can enhance cognitive abilities such as intelligence and attention (Gupta et al., 2018), while sad music often serves as an effective tool for emotional regulation and coping in challenging situations, as well as for improving attention (Gupta et al., 2023).

To achieve the study's objectives, we conducted a comparative microstate analysis across three conditions—baseline (BL), music (MUS), and post-music (PMS)—for each case while participants listened to happy and sad musical stimuli. We hypothesize that happy music will predominantly affect class D microstates, signifying enhanced attention during the experience of pleasant music. In contrast, sad music is expected to influence both class C and class D microstates, which are associated with self-referential processing (DMN) and attention, respectively.

## 2 Method

### 2.1 Participants

This study utilized two separate secondary datasets to investigate the effects of happy and sad classical music, respectively. The first dataset (Gupta et al., 2018) consisted of 20 participants with a mean age of 24.06 years (SD = 2.69), who listened to happy classical music. The second dataset (Gupta et al., 2023) consisted of 20 participants with a mean age of 22.14 years (SD = 3.68), who listened to sad classical music following an adverse experience of sad autobiographical recall (SAR) of a negative real-life event in which they experienced sadness such as feelings of loss, loneliness, misunderstanding, heartbreak, betrayal, loss of a loved one, etc. (Gupta et al., 2023; Hanser et al., 2016). Participants in both experiments were enrolled from a technology institute.

The methodology for these steps has been well-documented in the original study, and only relevant processing steps or modifications specific to this study are described below. To be eligible for the study, participants had to meet the criteria of having no formal or informal music training and being right-handed. The literature highlights differences in EEG microstates between musicians and non-musicians. Therefore, trained musicians were excluded from the analysis in the current study to maintain consistency. Exclusion criteria also encompassed hearing disorders, psychopathological diseases, neurological diseases, and recent usage of psychoactive drugs. Additionally, participants in the sad music experiment were screened out for any predisposition to depression. This precaution aimed to prevent the maladaptive use of sad music as a coping mechanism for emotion regulation in individuals prone to depression. To minimize potential confounding factors, only male participants were included in both experiments. This decision was based on previous observations of differences in biomarkers for cognitive (Neubauer and Fink, 2009) and emotional processes (Goshvarpour and Goshvarpour, 2019) between male and female participants (Whittle et al., 2011). The studies were duly approved by the Institutional Ethics Committee (IEC) involving human subjects of the Indian Institute of Technology, Kanpur (IEC Communication no: IITK/IEC/2019–20/I/18, IITK/IEC/2017–18 I/3). Throughout the entire study, adherence to relevant guidelines and regulations was strictly upheld.

### 2.2 Stimulus and experimental procedure

The experiments were conducted in a soundproof laboratory to minimize external interference. Participants were seated comfortably, with stereo speakers positioned symmetrically about 2 m away for free-field auditory stimulus delivery. The room was dimly lit to create a calm atmosphere and reduce distractions, ensuring auditory stimuli were the primary focus.

Indian classical music was selected as an experimental stimulus due to its proven effects on cognitive and emotional brain functions (Gupta et al., 2018, 2023). Researches show that Indian Ragas reduce stress, anxiety, and blood pressure (Kar et al., 2015; Siritunga et al., 2013), while enhancing life satisfaction and optimism (Gupta and Gupta, 2016). Previous EEG studies have demonstrated their ability to modulate neural activity (Gupta et al., 2018, 2023), making them ideal for exploring their impact on brain's microstates. The stimulus utilized for our investigation was performed by skilled professional musicians (Gupta et al., 2018, 2023).

The first experiment investigated the effects of listening to happy music. It comprised three distinct states: a baseline resting state (duration: 275 s), a music listening state involving participants attentively hearing the happy music with their eyes closed, and finally a post-music silence state (duration: 275 s). Raga Darbari segment (duration: 9 min and 53 s) was used as the happy musical stimulus. Participants also rated their mood on an 11-point Likert scale upon listening to Raga Darbari during the experiment.

The second experiment investigated the effects of listening to sad music during an adverse situation. It encompassed four distinct conditions of 9 min each. First, there was a baseline resting state. Following that, participants engaged in a SAR condition, where they recalled a personal episode that evoked sadness. Subsequently, participants listened to sad music. Finally, there was a post-music silence condition. The Mishra Raga Jogiya segment (duration: 8 min and 44 s) was used as the sad musical stimulus. During the baseline, sad music listening, and post music silence conditions, participants were instructed to maintain a calm seated position while focusing their gaze on a centrally printed cross displayed on a blank sheet of paper. However, during the SAR condition, the cross was substituted with a writing pad. In this condition, they were encouraged to vividly and in detail report the real-life episode that evoked feelings of sadness, encompassing experiences such as loss, loneliness, heartbreak, betrayal, etc. (Hanser et al., 2016) in the writing pad while supporting their elbow to minimize hand movements. Furthermore, participants were instructed to minimize any movement, including eye, head, and body movements, to minimize artifacts during the task while performing it in a natural manner.

Participants evaluated the vividness and reliving of autobiographical recall on a five-point scale. They also evaluated their mood on an 11-point Likert scale during the three states. Following the EEG experiment, participants completed a standard Self-Regulatory Goals Assessment questionnaire to asses self-regulatory goals upon sad raga listening. Additionally, they rated the efficiency of the sad musical stimulus in managing the SAR condition on an 11-point bidirectional scale with a range from –5 to +5.

### 2.3 EEG recording and preprocessing

In both studies, the EEG signals from the participants were recorded using a g.HIamp bio-signal amplifier (Guger Technologies, OG, Graz, Austria). The EEG data were recorded at a sampling frequency of 512 Hz, and it was collected from 32 scalp positions following the International 10-20 system. The impedance level was maintained below 5 Kohms. To ensure appropriate signal quality, the EEG data was band-pass filtered between 0.01 and 100 Hz. In addition, EEG data were also recorded from four electrooculography (EOG) positions, including the upper and lower right eye and the outer canthus locations of both eyes, to detect and eliminate any artifacts caused by eye blinks.

EEG preprocessing was performed using the EEGLAB toolbox (Delorme and Makeig, 2004). To enhance data processing, EEG data were down-sampled to a frequency of 256 Hz, and a high-pass filter with a 0.5 Hz cutoff was employed to eliminate any DC drift present in the signals. Visual inspection was performed to identify and mark any artifacts resulting from eye movements, muscle activity, or electrode movement. Bad electrodes were identified and interpolated to improve data quality. The EEG data were average-referenced. Independent Component Analysis (ICA) and SASICA (Semi-Automatic Selection of Independent Component Analysis) were employed to further eliminate artifacts caused by eye and muscle movements (Chaumon et al., 2015; Crespo-Garcia et al., 2008). EEG data were filtered to obtain the alpha band between 8 and 13 Hz. After excluding participants with high EEG artifacts and previous exposure to music, we were left with a total of 15 participants for each experiment, who were included in the subsequent microstate analysis.

### 2.4 Microstate analysis

We conducted a spatial k-means cluster analysis using the EEGLAB toolbox for each condition (Poulsen et al., 2018). The analysis utilized maps based on the local maxima of the GFP, which identifies time points characterized by the largest signal-to-noise ratio. The analysis did not consider the polarity of the maps. The microstate cluster analysis was conducted on the combined EEG data of all participants within each condition. Brain microstate maps are typically categorized into classes A, B, C, and D based on their topological orientations (Koenig et al., 1999). Specifically, microstate map A has a left-right orientation, map B shows a right-left orientation, map C displays an anterior-posterior orientation, and map D has a fronto-central maximum. This labeling convention has been consistently used in various studies (Michel and Koenig, 2018; Hu et al., 2023; Pal et al., 2021; Liu et al., 2021; Pascual-Marqui et al., 2014) (Supplementary Figures S2–S4). In our research, we followed this convention and classified the microstates into classes A, B, C, and D according to their topographical orientations as described initially by Koenig et al. (1999), in line with subsequent studies (Hu et al., 2023; Pal et al., 2021; Liu et al., 2021; Pascual-Marqui et al., 2014).

**Figure 1:**
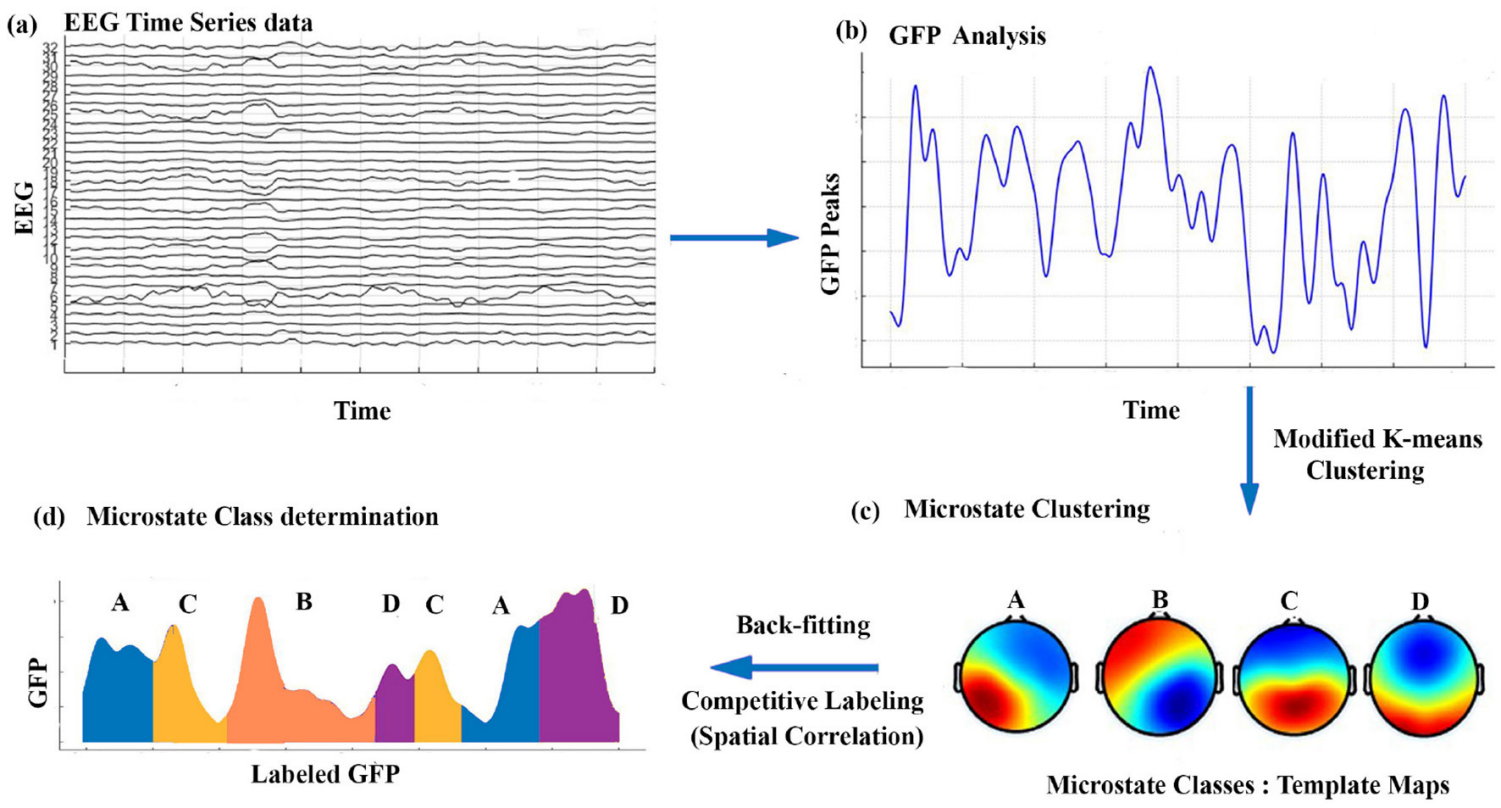
A schematic of the microstate analysis process: **(a)** preprocessed EEG data, **(b)** high SNR topographies extracted from GFP maxima, **(c)** clustering for reliable microstate map detection, and **(d)** mapping microstates back to EEG data, assigning each time point to a dominant state, followed by feature computation.

Additionally, we calculated the spatial correlation among the four microstates of the brain under different conditions. Once the maps were identified for each condition, they were applied to each participant's EEG data within that specific condition. Each frame of time in the EEG data was assigned to the template that exhibited the best spatial correlation match. This procedure produced a microstate sequence unique to each participant, and these sequences were subsequently employed to compute participant-specific microstate parameters for each condition. Figure 1 illustrates the microstate analysis procedure applied to each participant under each condition.

1. GFP: It serves as a reference-independent measure, representing the magnitude of the scalp electric field. GFP is equivalent to the spatial standard deviation of voltage amplitude and is typically measured in micro-volts (μV) (Murray et al., 2008; Skrandies, 1990).
2. GEV: This parameter quantifies the degree to which the selected template effectively represents the entire dataset. It is computed by summing the explained variances, with each value weighted according to the corresponding GFP at each time point (Murray et al., 2008).
  $${\text{GEV}}_{t}=\text{corr}{\left(s_{t},m_{l_{t}}\right)}^{2}\frac{GFP_{t}^{2}}{\sum_{t^{\prime }}^{T}GFP_{t^{\prime }}^{2}}$$
  In this context, GFP_*t*_ represents the global field power for the *t*^*th*^ time sample. The variable *s*_*t*_ denotes EEG data corresponding to the *t*^*th*^ time, *l*_*t*_ signifies the label of the microstate of *t*^*th*^ EEG data, *m*_*l*_*t*__ stands for the microstate map corresponding to the $l_{t}^{th}$, and *T* is the total time period.
3. Coverage: It represents the percentage of time frames in which a particular microstate is present, indicating the relative duration of its activation (Khanna et al., 2015; Murray et al., 2008).
4. Occurrence: The mean number of times the microstate is observed within a 1-s period. It reflects the tendency of intracortical sources to synchronize their activation and is measured in Hertz (Hz) (Khanna et al., 2015).
5. Duration: The mean temporal duration pertains to the average time span over which consecutive maps are attributed to the same microstate class (Khanna et al., 2015).

For correlation analysis during music listening, we divided the duration of the MUS condition into 10 segments. Microstates parameters calculated for each segment for each participant were utilized for correlation analysis. For comparative microstate analysis between BL, MUS, and PMS conditions, we selected 200 s of segment duration from each condition in both experiments (Supplementary Figure S1).

### 2.5 Statistical analysis

To investigate the effects of microstate class on parameters such as global explained variance (GEV), occurrence, duration, global field power (GFP), and coverage during music listening, a one-way repeated measures ANOVA was conducted using SPSS, with microstate class treated as a within-subject factor. Additionally, to examine the combined effects of microstate class and experimental conditions, a two-way repeated measures ANOVA was performed, considering both factors as within-subject variables.

The mean values of the above variables (*Post hoc* analysis) and the subjective questionnaire scores were compared using a two-tailed *t-*test at a significance level of (α) = 0.05, and false discovery rate (FDR) correction was applied to address issues related to multiple comparisons. Furthermore, correlation analyses were conducted between Class C and Class D microstates for parameters including GEV, coverage, and GFP during music listening.

## 3 Results

### 3.1 Experiment 1

#### 3.1.1 Microstate analysis for a happy Indian raga

**Figure 2:**
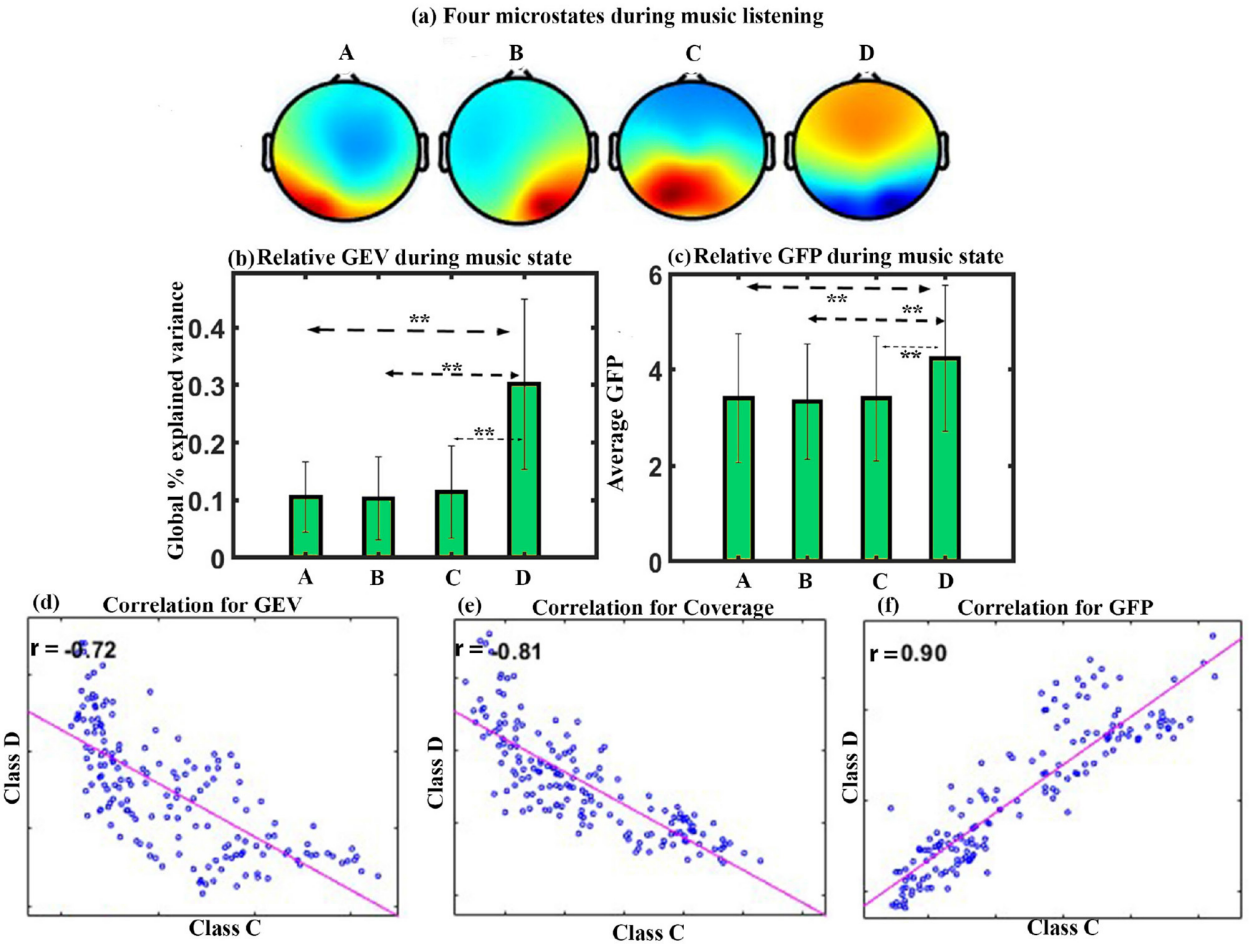
Microstate analysis for happy Indian raga. **(a)** Four EEG microstates underpinning music (MUS) conditions. **(b)** Relative GEV for each microstate class during music listening. **(c)** Relative GFP in each microstate class during music listening. **(d)** Correlation between the microstate class D and class C for GEV. **(e)** Correlation between the microstate class D and class C for Coverage. **(f)** Correlation between the microstate class D and class C for GFP (**FDR corrected, *p* < 0.05; error bars = 1 SD).

We performed microstate analysis for the full duration of Raga Darbari music. Figure 2a shows the four microstates underpinning raga darbari that explain 77.4% of GEV. The microstates are arranged according to the standard convention of classes A-D. The microstate maps were fitted back into the EEG data of the participants, yielding various parameters such as GEV, coverage, occurrence, duration, and inter-microstate transition probability.

**GEV**: We applied a one-way repeated ANOVA to examine the effect of microstate class on GEV. The results show a significant effect with a Greenhouse-Geisser correction (F_1.741, 24.379_ = 12.246, *p* < 0.001). The *post hoc* paired *t*-test shows that the microstate of class D is significantly higher compared to class A (t = 4.4643, df = 14, *p* < 0.005, d = 1.1527), class B (t = 4.8103, df = 14, *p* < 0.005, d = 1.2420), and class C (t = 3.3471, df = 14, *p* < 0.05, d = 0.8642) during happy music listening as shown in Figure 2b.

**GFP**: One-way repeated ANOVA with a Greenhouse-Geisser correction indicates a significant effect of microstate class on the GFP (F_1.797, 25.163_ = 28.452, *p* < 0.001). *Post hoc* paired *t*-test shows that class D microstate has significantly higher GFP than class A (t = 7.1884, df = 14, *p* < 0.001, d = 1.8560), class B (t = 8.0431, df = 14, *p* < 0.001, d = 2.0767), and class C (t = 5.1199, df = 14, *p* < 0.001, d = 1.3220) during happy music listening, as shown in Figure 2c. One-way repeated ANOVA effects of coverage, occurrence, duration, and inter-microstate transition probability were not significant. The results also showed a significant negative correlation between microstate class C and class D for GEV (r = –0.72, *p* < 0.001) as shown in Figure 2d, for coverage (r = –0.81, *p* < 0.001) as shown in Figure 2e, and positive correlation between microstate class C and class D for GFP (r = 0.9, *p* < 0.001) as shown in Figure 2f.

#### 3.1.2 Comparative microstate analysis among baseline resting state (BL), music (MUS), and post-music silence (PMS)

**Figure 3:**
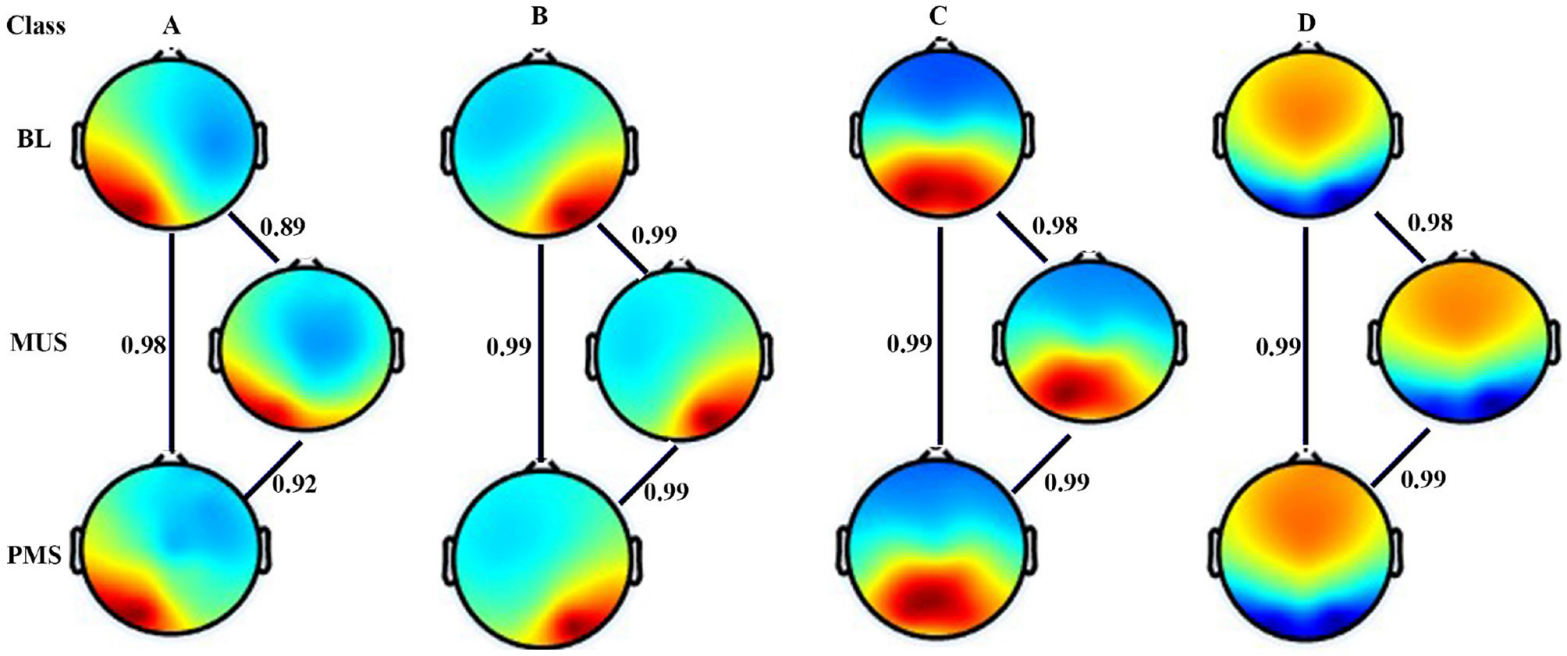
Microstate maps. Four EEG microstates under baseline (BL), music (MUS), and post-music silence (PMS) conditions. Spatial correlation between the corresponding microstate class across conditions.

Figure 3 shows four microstates that explained the variance (GEV) of 75.5%, 77.4%, and 74.43% during BL, MUS, and PMS conditions, respectively, for experiment 1. The underpinning microstates for the three conditions are arranged according to the standard convention. Results show strong spatial correlation of 0.9 among all the conditions for the corresponding microstate classes A-D (*p* < 0.001). To compare the conditions of BL, MUS, and PMS, 200 s of time duration for each condition was selected for further investigation (Supplementary Figure S1).

**Figure 4:**
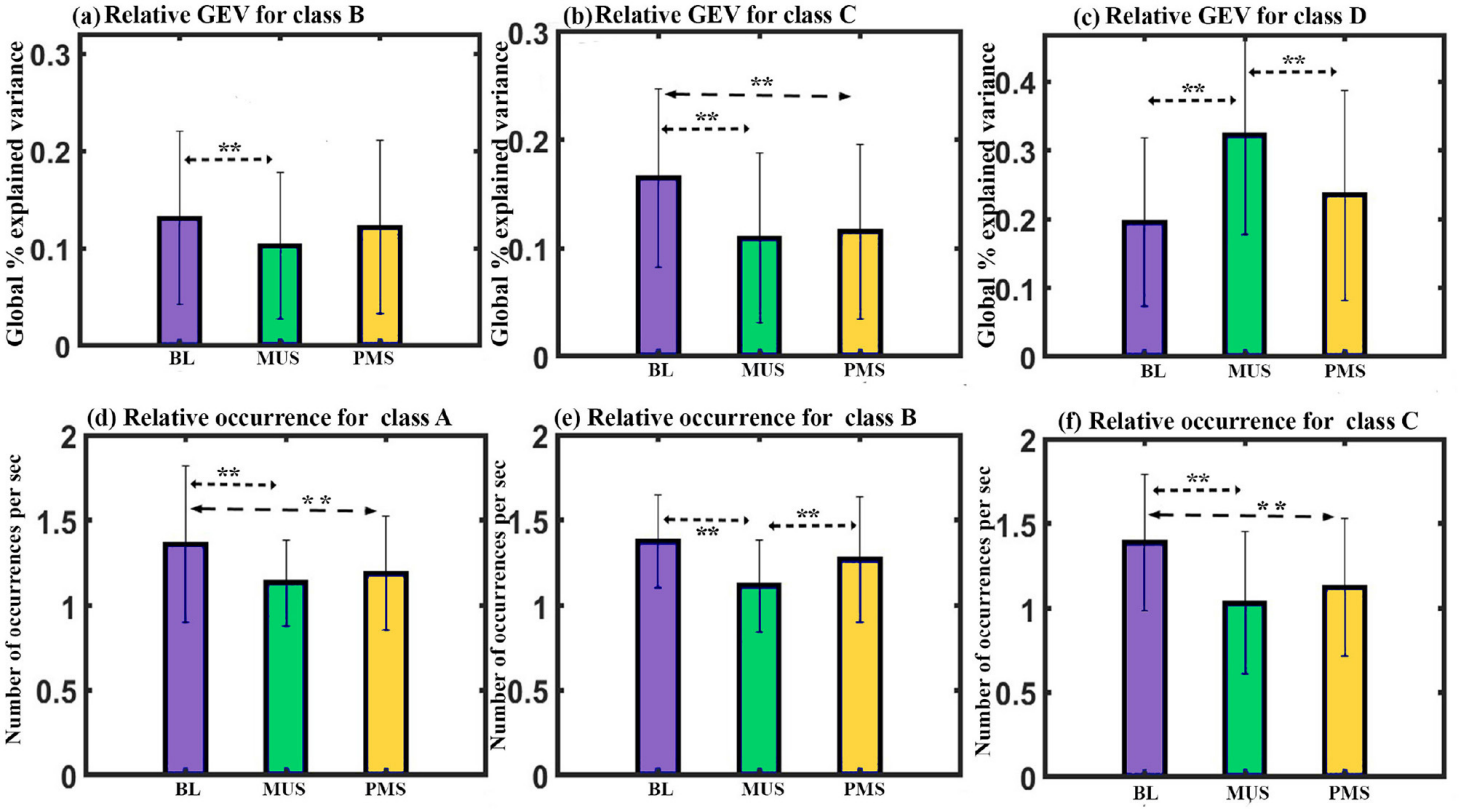
Microstates' properties. **(a)** Relative GEV of microstates class B across conditions. **(b)** Relative GEV of microstates class C across conditions. **(c)** Relative GEV of microstates class D across conditions. **(d)** Relative occurrence of microstates class A across conditions. **(e)** Relative occurrence of microstates class B across conditions. **(f)** Relative occurrence of microstates class C across conditions (**FDR corrected, *p* < 0.05; error bars = 1 SD).

**GEV**: A two-way repeated ANOVA with microstate class and condition as within factors shows a significant interaction with a Greenhouse-Geisser correction (F_2.933, 41.068_ = 7.474, *p* < 0.001). The one-way follow-up repeated ANOVA shows a significant effect on GEV of class B (F_2, 28_ = 5.015, *p* < 0.05), class C (F_2, 28_ = 12.960, *p* < 0.001), and class D (F_2, 28_ = 9.104, p = 0.001). Further *post hoc* paired *t*-test with FDR correction shows class B microstate during BL condition to have significantly higher GEV than class B during MUS (t = 3.5659, df = 14, *p* < 0.05, d = 0.9207) condition as shown in Figure 4a. Class C microstate during BL condition has significantly higher GEV than class C during MUS (t = 5.7033, df = 14, *p* < 0.0005, d = 1.4726) and PMS (t = 3.3379, df = 14, *p* < 0.05, d = 0.8618) conditions as shown in Figure 4b. The results also showed that the class D microstate during the MUS condition had a significantly enhanced GEV than that during BL (t = 3.4757, df = 14, *p* < 0.05, d = 0.8974) and PMS (t = 2.8331, df = 14, *p* < 0.05, d = 0.7315) condition as shown in Figure 4c.

**Occurrence**: A two-way repeated ANOVA with microstate class and condition as within factors yielded a significant interaction (F_6, 84_ = 4.995, *p* < 0.001). Follow-up one-way repeated ANOVA shows a significant effect on the occurrence of class A (F_2, 28_ = 5.263, *p* < 0.011), class B (F_2, 28_ = 5.943, *p* < 0.01), and class C (F_2, 28_ = 8.138, *p* < 0.01) microstates. Further *post hoc* paired *t*-test with FDR correction shows class A microstate during BL condition to have significantly higher occurrence compared to class A during MUS (t = 2.5521, df = 14, *p* < 0.05, d = 0.6589) and PMS (t = 2.6895, df = 14, *p* < 0.05, d = 0.6944) conditions as shown in Figure 4d. Class B microstate during the MUS condition has a significantly lower occurrence than class B during BL (t = –3.8205, df = 14, *p* < 0.01, d = –0.9865) and PMS (t = –2.2660, df = 14, *p* < 0.05, d = –0.5851) conditions as shown in Figure 4e. The results also showed that the class C microstate during the BL condition had a significantly increased occurrence compared to that during MUS (t = 4.2302, df = 14, *p* < 0.01, d = 1.0922) and PMS (t = 2.5806, df = 14, *p* < 0.05, d = 0.6663) conditions as shown in Figure 4f.

**Figure 5:**
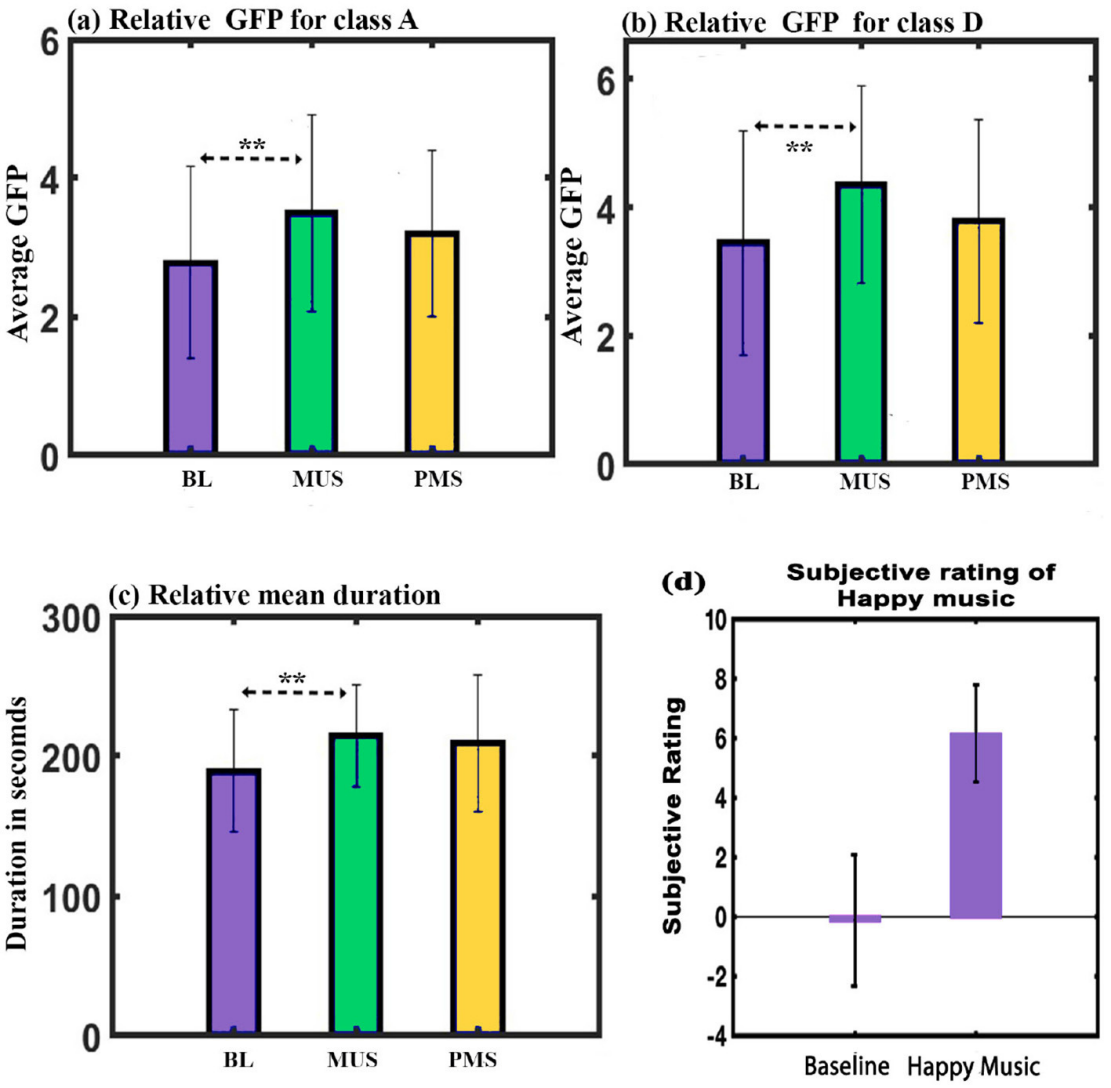
**(a)** Relative GFP of microstate class A across conditions. **(b)** Relative GFP of microstate class D across conditions. **(c)** Relative mean duration of all microstates across conditions. **(d)** Depicts the average subjective mood assessment before and after listening to music (**FDR corrected, *p* < 0.05; error bars = 1 SD).

**GFP**: A two-way repeated ANOVA with microstate class and condition as within factors resulted in a significant interaction effect (F_6, 84_ = 9.825, *p* < 0.001). Follow-up one-way repeated ANOVA shows a significant effect on GFP of class A (F_2, 28_ = 4.411, *p* < 0.05) and class D (F_2, 28_ = 5.484, p = 0.01) microstates. Further *post hoc* paired *t*-test with FDR correction shows class A microstate during MUS condition to have significantly higher GFP than class A during BL condition (t = 2.9839, df = 14, *p* < 0.05, d = 0.7704) as shown in Figure 5a. Class D microstate during MUS condition has significantly higher GFP than class D during BL condition (t = 3.8781, df = 14, *p* < 0.05, d = 1.0013) as shown in Figure 5b.

**Duration**: A two-way repeated ANOVA with microstate class and condition as within factors did not yield a significant interaction. However, we obtained a simple effect of conditions with a Greenhouse-Geisser correction (F_1.434, 41.016_ = 3.859, *p* = 0.05). Further *post hoc* paired *t*-test with FDR correction shows the mean value of the duration of all microstates during the MUS condition to have significantly higher compared to the BL condition (t = 3.2203, df = 14, *p* < 0.05, d = 0.8315) as shown in Figure 5c.

**Subjective ratings**: Raga darbari segment significantly expressed happiness (t = –9.5232, df = 14, *p* < 0.001, d = –2.4589)(Gupta et al., 2018) in the participants as shown in Figure 5d.

### 3.2 Experiment 2

#### 3.2.1 Microstate analysis for a sad Indian raga

**Figure 6:**
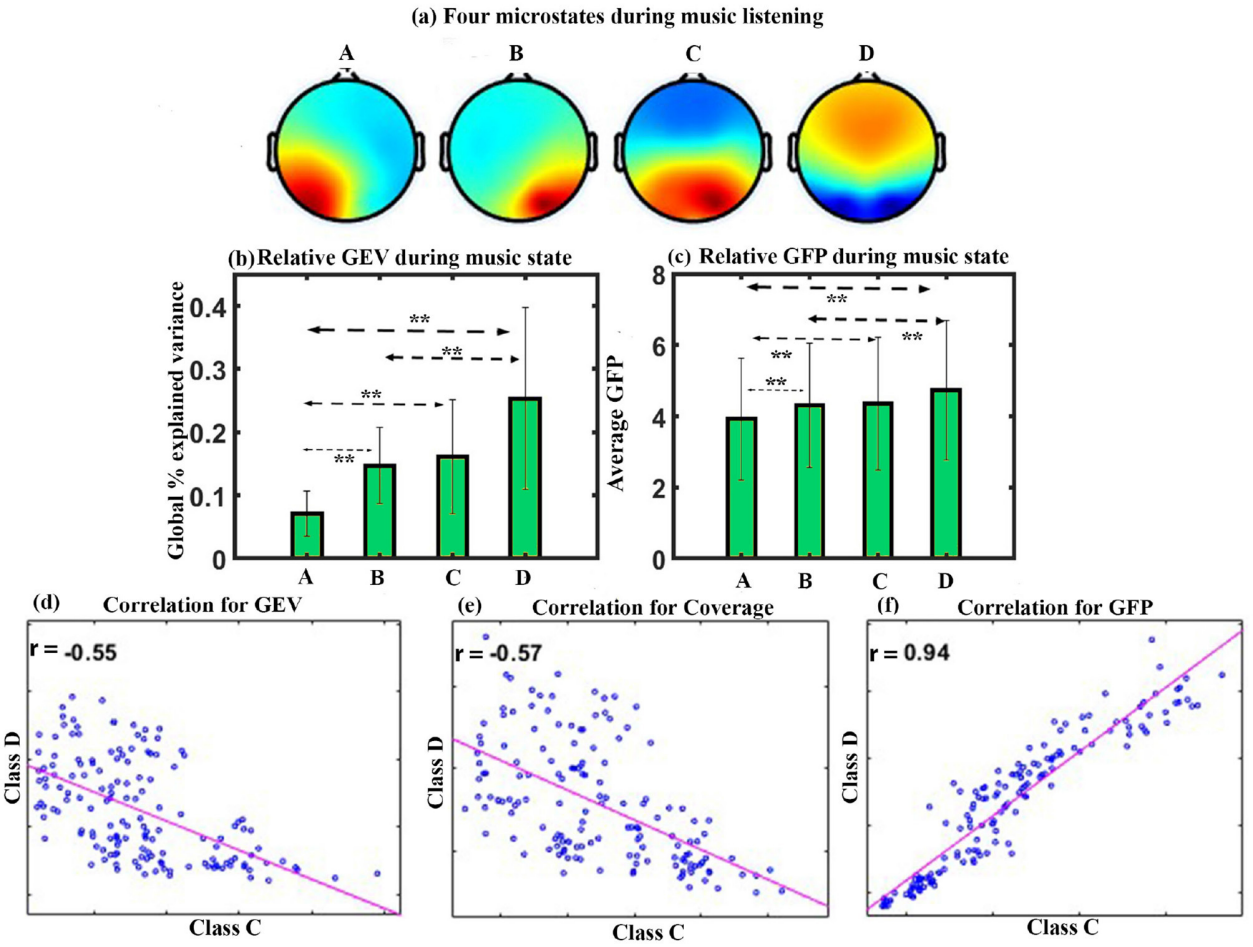
Microstate analysis for sad Indian raga. **(a)** Four EEG microstates under MUS conditions. **(b)** Relative GEV in each microstate class during music listening. **(c)** Relative GFP in each microstate class during music listening. **(d)** Correlation between the microstate class D and class C for GEV, **(e)** Correlation between the microstate class D and class C for Coverage, and **(f)** correlation between the microstate class D and class C for GFP (**FDR corrected, *p* < 0.05; error bars = 1 SD).

We conducted microstate analysis for the full duration of Raga Mishra jogiya. Figure 6a shows the four microstates underpinning the raga that explain 77% of GEV. The microstates are arranged according to the standard convention of class A-D. The microstate maps were fitted back into the EEG data of the participants, yielding various parameters such as GEV, coverage, occurrence, duration, and inter-microstate transition probability.

**GEV**: We applied a one-way repeated ANOVA to examine the effect of microstate class on GEV. The results show a significant effect with a Greenhouse-Geisser correction (F_1.656, 23.177_ = 7.719, *p* < 0.005). The *post hoc* paired *t*-test shows significantly higher presence of microstate of class D than class A (t = 4.3145, df = 14, *p* < 0.005, d = 1.1140) and class B (t = 2.4011, df = 14, *p* < 0.05, d = 0.6200). Class C and class B microstates are significantly higher as compared to class A microstate with (t = 3.3963, df = 14, *p* < 0.01, d = 0.8769) and (t = 5.4788, df = 14, *p* < 0.005, d = 1.4146), respectively, during sad music listening as shown in Figure 6b.

**GFP**: One-way repeated ANOVA with a Greenhouse-Geisser correction indicates a significant effect of microstate class on the GFP (F_1.911, 26.751_ = 11.126, *p* < 0.001). *Post hoc* paired *t*-test shows that the microstate of class D is significantly higher in GFP than class A (t = 5.2858, df = 14, *p* < 0.001, d = 1.3648) and class B (t = 3.6980, df = 14, *p* < 0.005, d = 0.9548). Class C and class B also have significantly higher GFP than class A with (t = 3.4829, df = 14, *p* < 0.005, d = 0.8993) and (t = 5.1187, df = 14, *p* < 0.001, d = 1.3217), respectively, during sad music listening as shown in Figure 6c. We did not observe a significant effect of coverage, occurrence, duration, and inter-microstate transition probability. The results also showed a significant negative correlation between microstate class C and class D for GEV (r = –0.55, *p* < 0.001) as shown in Figure 6d, for coverage (r = –0.57, *p* < 0.001) as shown in Figure 6e, and a positive correlation between microstate class C and class D for GFP (r = 0.94, *p* < 0.001) as shown in Figure 6f.

#### 3.2.2 Comparative microstate analysis between BL, MUS, and PMS

**Figure 7:**
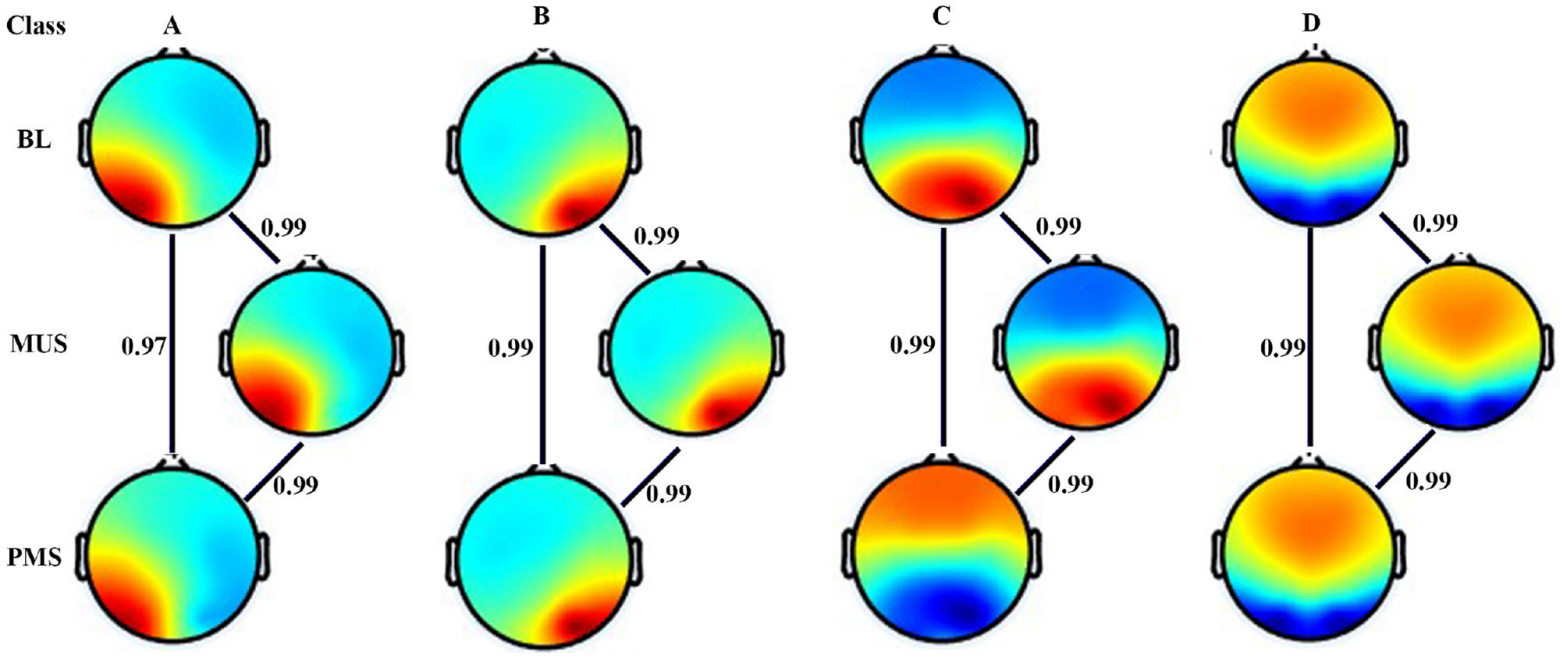
Microstate maps. Four EEG microstates under BL, MUS, and PMS conditions. Spatial correlation between the corresponding microstate class across conditions.

Figure 7 shows four microstates that explained the variance (GEV) of 77.95%, 77.77%, and 76.98% during BL, MUS, and PMS conditions, respectively, for experiment 2. The underpinning microstates for the three conditions are arranged according to the standard convention. Results show strong spatial correlation of 0.9 among all the conditions for the corresponding microstate classes A–D (*p* < 0.001). To compare the conditions of BL, MUS, and PMS, 200 s duration was selected for further investigation (Supplementary Figure S1).

**GEV**: A two-way repeated ANOVA with microstate class and condition as within factors shows a significant interaction with a Greenhouse-Geisser correction (F_3.1, 43.406_ = 3.251, *p* < 0.05). Follow-up one-way repeated ANOVA shows a significant effect on GEV of class C (F_2, 28_ = 4.036, *p* < 0.05) and class D (F_2, 28_ = 6.236, *p* < 0.01) microstates.

**Figure 8:**
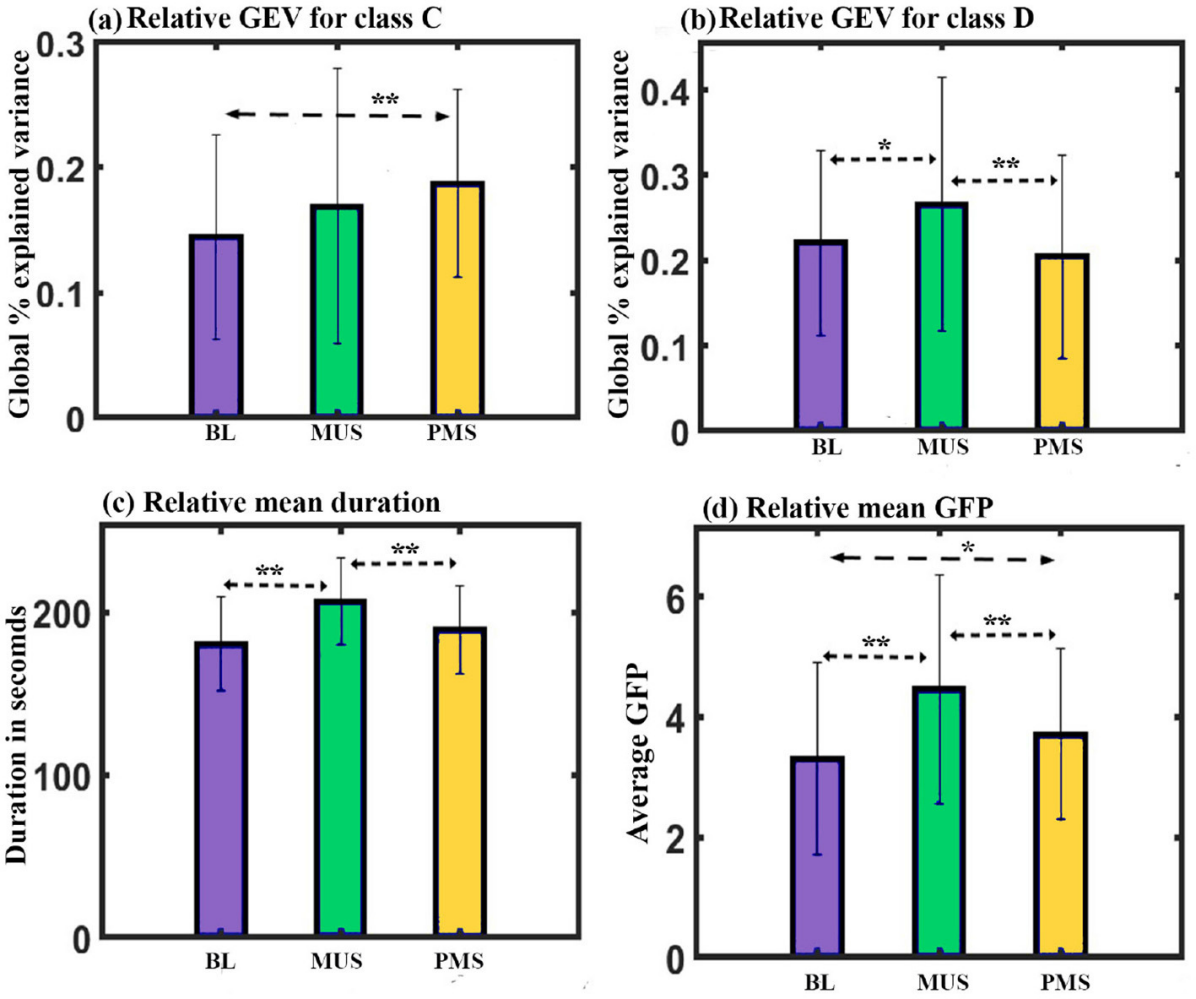
Microstate parameters. **(a)** Relative GEV of microstates class C across conditions. **(b)** Relative GEV of microstates class D across conditions. **(c)** Relative mean duration of all microstates across conditions. **(d)** Relative mean GFP of all microstates across conditions (*uncorrected,**FDR corrected, *p* < 0.05; error bars = 1 SD).

Further *post hoc* paired *t*-test with FDR correction shows class C microstate during PMS condition to have significantly higher GEV than class C during BL (t = 3.4570, df = 14, *p* < 0.05, d = 0.8994) condition as shown in Figure 8a. Class D microstate during MUS condition has significantly higher GEV than class D during BL [t = 2.0696, df = 14, *p* < 0.05 (uncorrected), d = 0.5344] and PMS (t = 3.9638, df = 14, *p* < 0.01, d = 1.0234) conditions as shown in Figure 8b.

**Duration**: Two-way repeated ANOVA with microstate class and condition as within factors did not show a significant interaction. However, we obtained a simple effect of conditions (F_2, 84_ = 12.702, *p* < 0.001). Further *post hoc* paired *t*-test with FDR correction shows mean value of the duration of all microstate during MUS condition to be significantly higher than BL (t = 5.4489, df = 14, *p* < 0.001, d = 1.4069) and PMS conditions (t = 4.2820, df = 14, *p* < 0.01, d = 1.1056) as shown in Figure 8c.

**GFP**: We administered a two-way repeated ANOVA with microstate class and condition as within factors. Although the interaction was not significant, we obtained the simple effect of conditions (F_2, 28_ = 11.220, *p* < 0.001). Further *post hoc* paired *t*-test with FDR correction shows mean GFP of all microstate during MUS condition to be significantly higher compared to BL condition (t = 4.0834, df = 14, *p* < 0.01, d = 1.0543) and PMS condition (t = 2.9317, df = 14, *p* < 0.05, d = 0.7570) as shown in Figure 8d.

**Figure 9:**
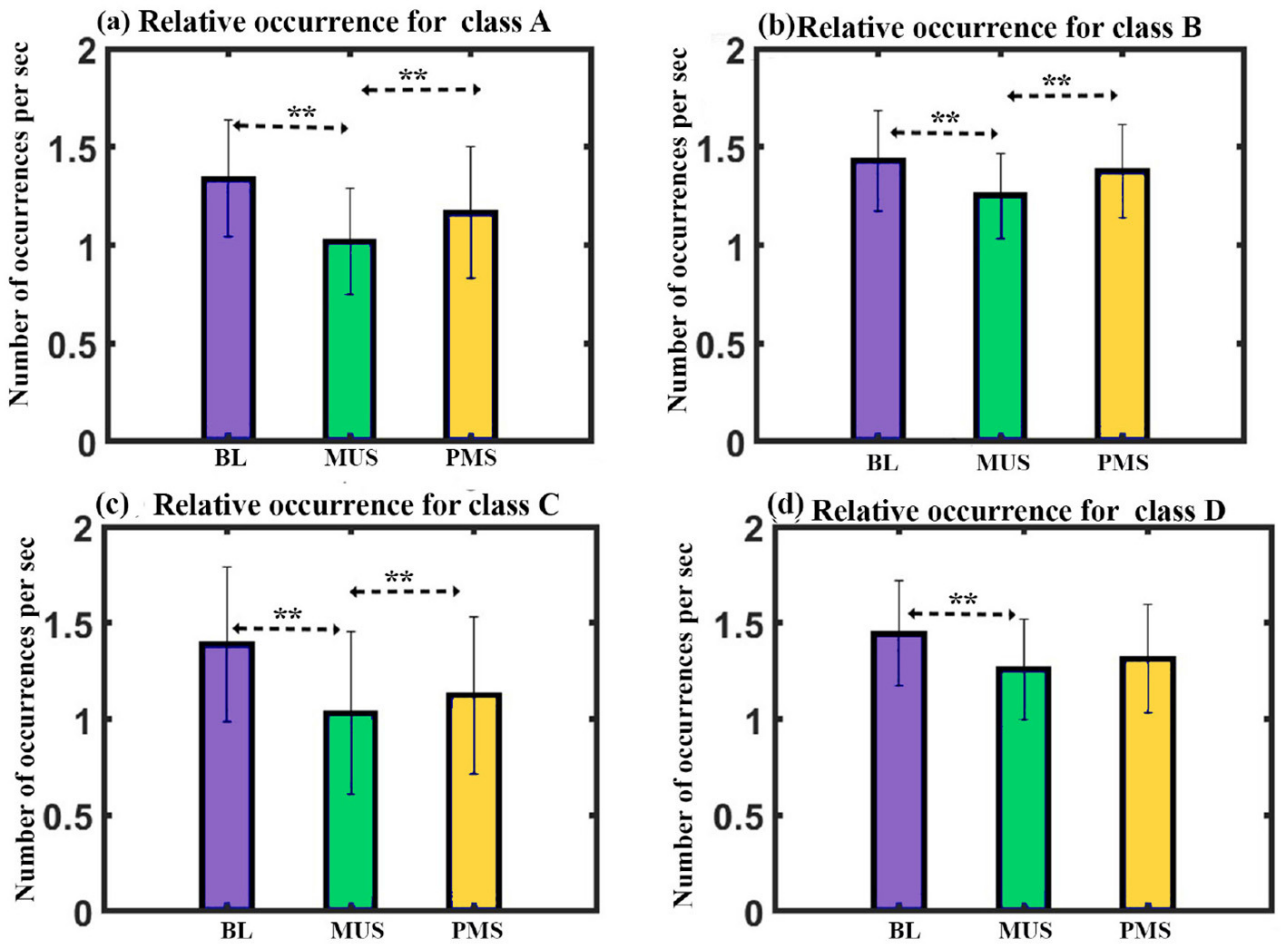
Microstate parameters. **(a)** Relative occurrence of microstates class A across conditions. **(b)** Relative occurrence of microstates class B across conditions. **(c)** Relative occurrence of microstates class C across conditions. **(d)** Relative occurrence of microstates class D across conditions (**FDR corrected, *p* < 0.05; error bars = 1 SD).

**Occurrence**: We administered a two-way repeated ANOVA with microstate class and condition as within factors. The results show a significant interaction with a Greenhouse-Geisser correction (F_3.299, 46.188_ = 3.122, *p* < 0.05). The one-way follow-up repeated ANOVA shows a significant effect on the occurrence of class A with a Greenhouse-Geisser correction (F_1.369, 19.164_ = 9.047, *p* < 0.005), class B (F_2, 28_ = 8.894, *p* = 0.001), class C (F_2, 28_ = 10.730, *p* < 0.001), and class D (F_2, 28_ = 5.313, *p* < 0.05). Further *post hoc* paired *t*-test with FDR correction shows class A microstate during MUS condition to have significantly lower occurrence than class A during BL (t = –3.6758, df = 14, *p* < 0.01, d = –0.9491) and PMS (t = –3.4482, df = 14, *p* < 0.01, d = –0.8903) conditions as shown in Figure 9a. The class B microstate during the MUS condition has significantly lower occurrence compared to class B during BL (t = –3.9214, df = 14, *p* < 0.01, d = –1.0125) and PMS (t = –4.1434, df = 14, *p* < 0.01, d = –1.0698) conditions as shown in Figure 9b. Class C microstate during MUS condition to have significantly lower occurrence than class C during BL (t = –3.7033, df = 14, *p* < 0.01, d = –0.9562) and PMS (t = –4.1552, df = 14, *p* < 0.01, d = –1.0729) conditions as shown in Figure 9c. The class D microstate during the MUS condition has significantly lower occurrence than class D during BL (t = –3.0188, df = 14, *p* < 0.05, d = –0.7795) as shown in Figure 9d.

**Figure 10:**
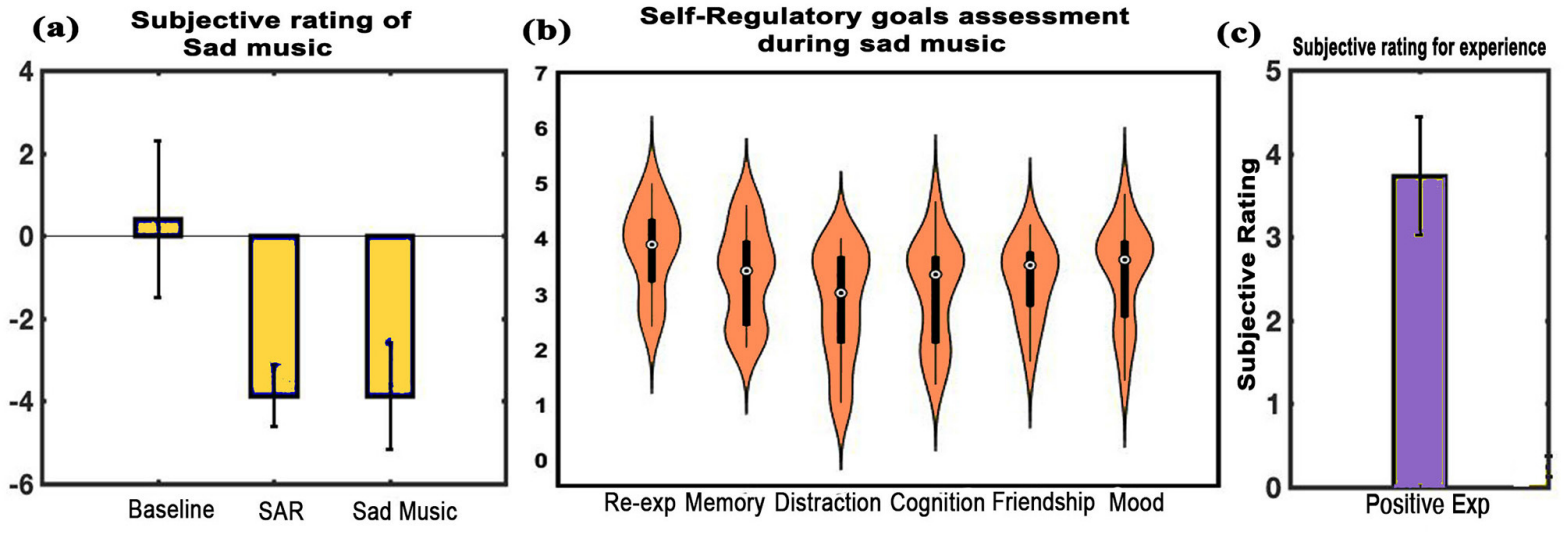
Subjective ratings. **(a)** Depicts average rating of the experiences across all states: BL, SAR, and listening to sad music, **(b)** shows the subjective ratings for self-regulatory goals during MUS condition, and **(c)** shows the average assessment of sad music listening. Findings indicate a positive experience while listening to sad music. Error bars represent one standard deviation (error bars = 1 SD).

**Subjective ratings**: The subjective ratings of memories recalled during SAR revealed mean scores of 4.2 (SD = 0.67) for vividness, 4.13 (SD = 0.74) for reliving, and 14.2 months (SD = 10.3) for the age of the memory. Figure 10a shows mood assessment by the participants with a mean score of 3.9 (SD = 0.7) during the SAR state and 3.9 (SD = 1.3) during sad music listening, compared to the baseline mean score of 0.4 (SD = 1.9). The differences were significant for both SAR (t = –8.663, df = 14, *p* < 0.001, d = –2.236) and sad music (t = –6.094, df = 14, *p* < 0.001, d = –1.5735) when compared to the BL state. No significant difference was found between the SAR and sad music conditions. Re-experiencing emotions was the predominant self-regulatory goal during sad music, with a mean of 3.7917 (SD = 0.7858). Other self-regulatory goals observed included a mean of 3.333 (SD = 0.8772) for memory, 2.7778 (SD = 1.0209) for distraction, 3.0444 (SD = 0.9666) for cognition, and 3.2333 (SD = 0.6974) for friendship (Figure 10b). Participants also unanimously reported positive experiences upon listening to sad music, with a mean score of 3.733 (SD = 0.7037), post SAR as shown in Figure 10c.

## 4 Discussion

Music has been recognized for its ability to influence emotions and cognitive processes. Happy music has been found to boost intelligence (Rauscher et al., 1993; Rideout and Laubach, 1996) and attention (Putkinen et al., 2017; Markovic et al., 2017; Jäncke et al., 2015), while sad music has been used to regulate emotions and cope with challenging situations (Van den Tol et al., 2016; Van den Tol and Edwards, 2013; Hanser et al., 2016). However, the specific brain microstates associated with these effects have not been fully understood. This study aimed to comparatively investigate the underlying microstates that contribute to the observed effects when listening to happy and sad Indian raga, in relation to BL and PMS conditions.

The first experiment investigated the effect of happy music listening. The subjective assessment shows that the musical stimulus successfully induced a moderate degree of happiness in the participants (Figure 5d). Furthermore, we obtained four microstates that explained the variance (GEV) of 75.5, 77.4, and 74.43 during BL, MUS, and PMS conditions, respectively. These findings are illustrated in Figures 2a, 3. The results revealed that the spatial distribution of these four microstates resembled those of the four classical microstates (Wang et al., 2021; Hu et al., 2023; Pascual-Marqui et al., 2014; Gu et al., 2022), including earlier microstate studies involving musical stimulus (Hu et al., 2023; Gupta et al., 2025).

The analysis of GEV and GFP during the course of happy music listening indicates that class D microstate exhibits significantly higher presence and increased electrical activity than all other microstates (Figures 1b, c). Furthermore, the analysis of GEV and coverage for correlation between class C and class D microstates demonstrates a robust negative association, consistent with previous research (Braboszcz and Delorme, 2011) (Figures 2d, e). It is worth noting that class C microstate is associated with mind-wandering, especially self-referential thoughts and processes, while class D microstate is linked to attention, respectively (Khanna et al., 2015; Michel and Koenig, 2018; Koenig et al., 2002; Tarailis et al., 2023). This suggests that listening to happy music is linked with heightened attention, and when attention is heightened (class D), mind-wandering (including self-referential thoughts and processes) tends to be diminished during the course of listening to happy music. Additionally, we observed a strong positive correlation between the two microstates for GFP, as shown in Figure 2f. This suggests that, while the electrical activity of both microstates exhibits a positive correlation during the course of listening to happy music, there is a negative correlation between their relative presence. The findings align with previous research involving Raga Darbari, which indicated enhanced attention and decreased interference from unwanted noise during the music listening experience (Gupta et al., 2018).

Furthermore, a comparative microstate analysis was conducted across three conditions: BL, MUS, and PSM. The analysis of GEV revealed a significantly greater presence of the class D microstate in the MUS condition than the other conditions (Figure 4c). Both the MUS and PSM conditions also revealed a significantly reduced presence of class C microstate than the BL condition (Figure 4b). The findings show that a happy music listening state is characterized by enhanced attention and diminished mind-wandering. This is further supported by the lower presence of the class B microstate during the MUS condition as compared to BL condition (Figure 4a). Class B microstate has been linked to scenes and self-visualization (Bréchet et al., 2019; Tarailis et al., 2023), thereby indicating that mind-wandering might be reduced upon listening to happy music. However, further studies are needed to ascertain it. The results obtained in our study align with previous research on music (Gupta et al., 2018, 2025). GFP analysis revealed that the music listening condition (MUS) exhibited enhanced electrical activity than the baseline condition for the classes A and D microstates (Figures 5a, b). Increased GFP for class D indicates a more activated attention state, consistent with the above findings. On the other hand, the increase in GFP for class A suggests that auditory processing is enhanced during music listening than the baseline condition. These results align with a previous study that demonstrated an increased level of music awareness during the listening of happy music (Taruffi et al., 2017).

The second experiment focused on the effect of sad music listening. Participants' subjective mood assessments revealed an increased sad state during sad music listening (Figure 10a). Although sadness was experienced in both the SAR state and sad MUS state, the self-regulatory questionnaire indicated a qualitative variation in the nature of this sadness. The questionnaire revealed that the sad musical excerpts facilitated the achievement of various self-regulatory goals, such as re-experiencing past emotions, enhancing mood, and evoking memories (Figure 10b). Additionally, the results indicated that listening to sad music post adverse experience resulted in an overall positive experience (Figure 10c), aligning with findings from previous studies (Van den Tol et al., 2016; Van den Tol and Edwards, 2013; Hanser et al., 2016).

We further obtained four microstates that explained variance (GEV) of 77.95, 77.77, and 76.98 during BL, MUS, and PMS conditions, respectively. These results are depicted in Figures 6A, 7. The results revealed that the spatial distribution of these four microstates resembled the classical four microstates (Wang et al., 2021; Hu et al., 2023; Pascual-Marqui et al., 2014; Gu et al., 2022), including earlier microstate studies involving musical stimulus (Hu et al., 2023; Gupta et al., 2025). During the course of sad music listening, analyses of GEV and GFP indicate that class A microstate exhibits significantly lower presence and decreased electrical activity than all other microstates (Figures 6b, c).

Additionally, we conducted a comparative microstate analysis across three conditions: BL, MUS, and PSM. The GEV analysis showed that the presence of class C microstate during the PMS condition was significantly higher than the BL condition, as shown in Figure 8a. Additionally, the presence of a class D microstate was significantly higher during the MUS condition than the BL and PMS states (Figure 8b).

It is worth noting that the phenomenon of mind-wandering during sad music listening differs from ordinary mind-wandering, and is distinguished by its melancholic yet pleasurable nature (Gupta et al., 2023; Taruffi and Koelsch, 2014; Sachs et al., 2015). It involves the emergence of spontaneous, self-referential thoughts, emotions, and cognitive processes (Gupta et al., 2023; Taruffi and Koelsch, 2014; Sachs et al., 2015). This is also in alignment with the results obtained in the subjective assessment. Thus, the enhanced presence of class C and class D microstates as a result of listening to sad music signifies an enhanced process of mind-wandering, especially self-referential and attention, respectively. These findings are consistent with previous studies (Gupta et al., 2023; Van den Tol and Edwards, 2013; Van den Tol et al., 2016).

Furthermore, the increased presence of class B microstate during sad music listening might indicate the involvement of scene and self-visualization with self-referential thoughts and memories during sad music listening. This aligns with earlier research (Bréchet et al., 2019; Gupta et al., 2023; Van den Tol and Edwards, 2013; Van den Tol et al., 2016); however, more investigations are needed to ascertain the fact.

The duration and GFP analysis show that regardless of the microstate class, the mean duration and GFP of microstates were higher during the MUS condition than the BL and PMS states, as shown in Figures 8c, d. This suggests that during sad music listening, there is a tendency for the brain microstates to persist for a longer duration with enhanced electrical activity.

Furthermore, the occurrence analysis showed that the frequency of occurrence for microstates was lower than other states during the MUS state, suggesting that the music state had a lower occurrence rate for microstates but with longer duration and larger GFP (Figures 9a–d).

Moreover, analyses of GEV and coverage for correlation between class C and class D microstates during the course of listening to sad music reveal a moderate negative correlation. It is important to note that this relationship explains only a small amount of variance in the data, as indicated by low R-squared values (0.3025 and 0.3249), as shown in Figures 6d, e. This suggests that there are other factors and parameters that contribute to the unexplained variance in the data. Future investigations should explore these additional factors and parameters. However, different relationships between class C and class D microstates (for GEV and coverage) during happy and sad music are consistent with earlier studies and likely highlight the differences in the nature of mind-wandering (self-referential) process (class C microstates) between them (Taruffi et al., 2017). We also observed a strong positive correlation between the two microstates (class C and class D) in terms of GFP, as shown in Figure 6f. This suggests that the electrical activity of both microstates is enhanced during the course of listening to sad music and is in line with the happy music analysis.

**Figure 11:**
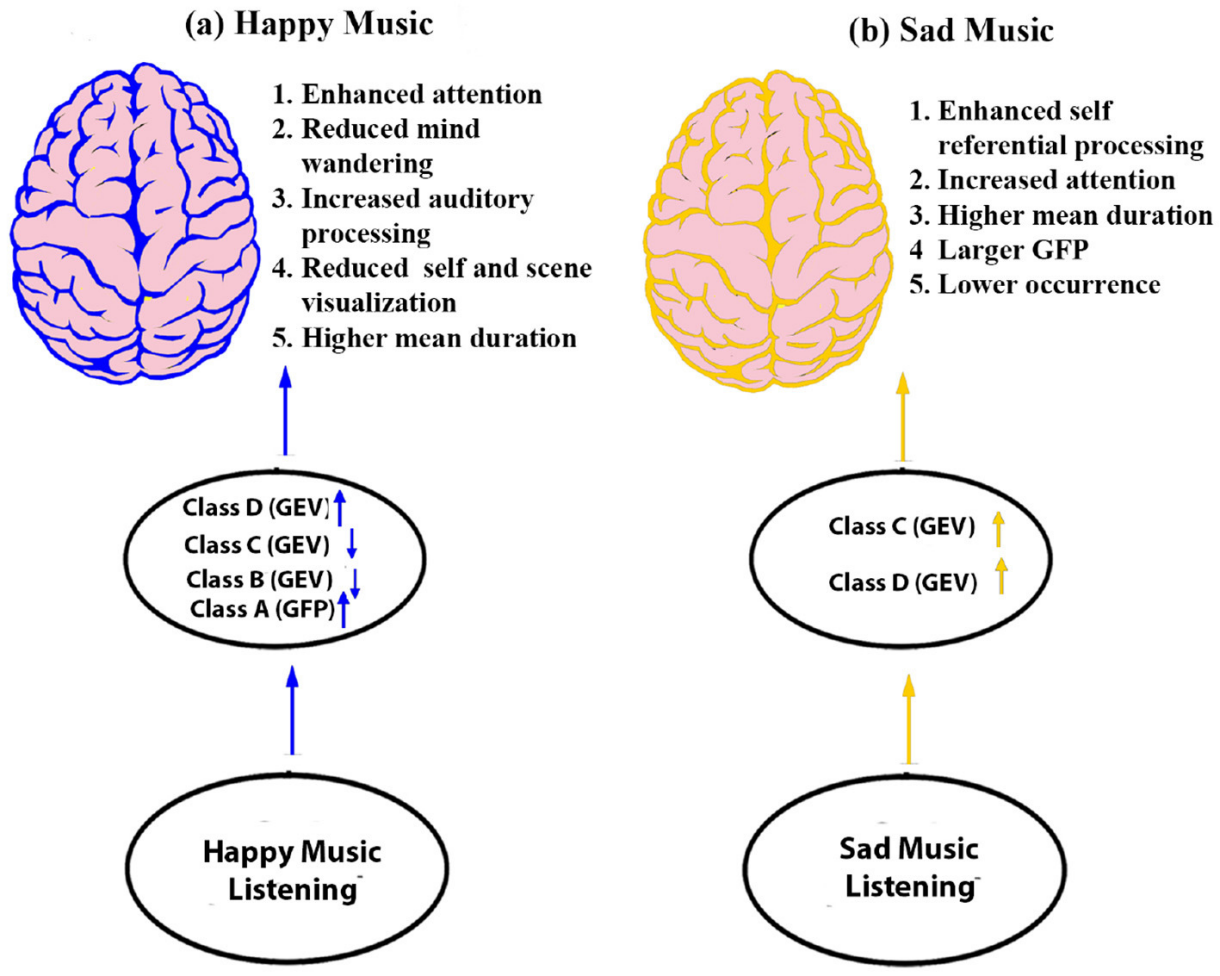
Schematic model illustrating the brain's responses during happy and sad music listening: **(a)** Happy music stimulates attention and reduces mind-wandering (depicted by blue color). **(b)** Sad music stimulates brain regions associated with attention, mind-wandering, particularly self-referential processes (depicted by yellow color).

In summary (Figure 11), the present study underscores the impact of happy and sad music on various mental processes, particularly in modulating brain microstates. The key findings indicate that listening to music leads to longer microstate duration and improved attention. Furthermore, happy music specifically reduces mind-wandering, fostering sustained focus, whereas sad music enhances self-referential processing, aiding in self-regulation during emotionally challenging situations.

This study's findings pave the way for personalized music therapy, cognitive training, and mental health interventions for conditions such as ADHD, depression, and anxiety. Music's impact on attention and self-regulation can enhance workplace productivity, education, and rehabilitation. Additionally, AI-driven adaptive music systems could tailor recommendations based on cognitive states. These insights have broad applications in healthcare, technology, and performance enhancement.

## 5 Limitations

While this study offers valuable insights, several limitations warrant further exploration. First, it focused on specific music genres, and incorporating a wider range of musical styles could deepen our understanding of music-induced brain dynamics. Additionally, the study was limited to male participants, underscoring the need for future research to examine potential gender differences. Furthermore, it did not consider how varying intensities of happiness and sadness influence microstates, particularly classes C and D. The lack of real-time subjective assessments of attention and mind-wandering also restricts insights into moment-to-moment cognitive fluctuations during music listening.

Future research utilizing a dense montage system with 64+ electrodes and source localization analysis could provide a more precise understanding of the neural mechanisms underlying microstate changes, particularly in differentiating self-referential processes during sad music listening. Age can be a crucial factor, as it may influence both neural processing and microstate dynamics. Thus, the current findings need to be validated across different age groups. Furthermore, Studies 1 and 2 were conducted on separate sample groups. Future research using the same sample group for both musical stimuli would allow for a more detailed comparative analysis of microstate features specific to happy and sad music listening. Addressing these gaps will contribute to a more comprehensive understanding of music's effects on brain function and its therapeutic applications.

## Data Availability

The raw data supporting the conclusions of this article will be made available by the authors, without undue reservation.

## References

[B1] Al Zoubi O. Mayeli A. Tsuchiyagaito A. Misaki M. Zotev V. Refai H.. (2019). EEG microstates temporal dynamics differentiate individuals with mood and anxiety disorders from healthy subjects. Front. Hum. Neurosci. 13:56. 10.3389/fnhum.2019.00056 30863294 PMC6399140

[B2] Andrews-Hanna J. R. Reidler J. S. Huang C. Buckner R. L. (2010a). Evidence for the default network's role in spontaneous cognition. J. Neurophysiol. 104, 322–335. 10.1152/jn.00830.2009 20463201 PMC2904225

[B3] Andrews-Hanna J. R. Reidler J. S. Sepulcre J. Poulin R. Buckner R. L. (2010b). Functional-anatomic fractionation of the brain's default network. Neuron 65, 550–562. 10.1016/j.neuron.2010.02.005 20188659 PMC2848443

[B4] Braboszcz C. Delorme A. (2011). Lost in thoughts: neural markers of low alertness during mind wandering. Neuroimage 54, 3040–3047. 10.1016/j.neuroimage.2010.10.008 20946963

[B5] Brattico E. Alluri V. Bogert B. Jacobsen T. Vartiainen N. Nieminen S.. (2011). A functional mri study of happy and sad emotions in music with and without lyrics. Front. Psychol. 2:308. 10.3389/fpsyg.2011.00308 22144968 PMC3227856

[B6] Bréchet L. Brunet D. Birot G. Gruetter R. Michel C. M. Jorge J. (2019). Capturing the spatiotemporal dynamics of self-generated, task-initiated thoughts with EEG and fMRI. Neuroimage 194, 82–92. 10.1016/j.neuroimage.2019.03.029 30902640

[B7] Cantero J. L. Atienza M. Salas R. M. Gómez C. M. (1999). Brain spatial microstates of human spontaneous alpha activity in relaxed wakefulness, drowsiness period, and rem sleep. Brain Topogr. 11, 257–263. 10.1023/A:1022213302688 10449257

[B8] Chaumon M. Bishop D. V. Busch N. A. (2015). A practical guide to the selection of independent components of the electroencephalogram for artifact correction. J. Neurosci. Methods 250, 47–63. 10.1016/j.jneumeth.2015.02.025 25791012

[B9] Chen J. Li H. Ma L. Bo H. Soong F. Shi Y. (2021). Dual-threshold-based microstate analysis on characterizing temporal dynamics of affective process and emotion recognition from EEG signals. Front. Neurosci. 15:689791. 10.3389/fnins.2021.689791 34335165 PMC8318040

[B10] Christoff K. Gordon A. M. Smallwood J. Smith R. Schooler J. W. (2009). Experience sampling during fMRI reveals default network and executive system contributions to mind wandering. Proc. Nat. Acad. Sci. 106, 8719–8724. 10.1073/pnas.0900234106 19433790 PMC2689035

[B11] Coll S. Y. Vuichoud N. Grandjean D. James C. E. (2019). Electrical neuroimaging of music processing in pianists with and without true absolute pitch. Front. Neurosci. 13:142. 10.3389/fnins.2019.00142 30967751 PMC6424903

[B12] Crespo-Garcia M. Atienza M. Cantero J. L. (2008). Muscle artifact removal from human sleep EEG by using independent component analysis. Ann. Biomed. Eng. 36, 467–475. 10.1007/s10439-008-9442-y 18228142

[B13] Custo A. Van De Ville D. Wells W. M. Tomescu M. I. Brunet D. Michel C. M. (2017). Electroencephalographic resting-state networks: source localization of microstates. Brain Connect. 7, 671–682. 10.1089/brain.2016.0476 28938855 PMC5736178

[B14] Delorme A. Makeig S. (2004). EEGlab: an open source toolbox for analysis of single-trial EEG dynamics including independent component analysis. J. Neurosci. Methods 134, 9–21. 10.1016/j.jneumeth.2003.10.009 15102499

[B15] Férat V. Seeber M. Michel C. M. Ros T. (2022). Beyond broadband: towards a spectral decomposition of electroencephalography microstates. Hum. Brain Mapp. 43, 3047–3061. 10.1002/hbm.25834 35324021 PMC9188972

[B16] Ford J. H. Addis D. R. Giovanello K. S. (2011). Differential neural activity during search of specific and general autobiographical memories elicited by musical cues. Neuropsychologia 49, 2514–2526. 10.1016/j.neuropsychologia.2011.04.032 21600227 PMC3137744

[B17] Goshvarpour A. Goshvarpour A. (2019). EEG spectral powers and source localization in depressing, sad, and fun music videos focusing on gender differences. Cogn. Neurodyn. 13, 161–173. 10.1007/s11571-018-9516-y 30956720 PMC6426927

[B18] Gu F. Gong A. Qu Y. Xiao H. Wu J. Nan W.. (2022). Research on top archer's EEG microstates and source analysis in different states. Brain Sci. 12:1017. 10.3390/brainsci12081017 36009079 PMC9405655

[B19] Gupta A. Bhushan B. Behera L. (2018). Short-term enhancement of cognitive functions and music: a three-channel model. Sci. Rep. 8, 1–12. 10.1038/s41598-018-33618-1 30341361 PMC6195580

[B20] Gupta A. Bhushan B. Behera L. (2023). Neural response to sad autobiographical recall and sad music listening post recall reveals distinct brain activation in alpha and gamma bands. PLoS ONE 18:e0279814. 10.1371/journal.pone.0279814 36607985 PMC9821717

[B21] Gupta A. Srivastava C. K. Bhushan B. Behera L. (2025). A comparative study of EEG microstate dynamics during happy and sad music videos. Front. Hum. Neurosci. 18:1469468. 10.3389/fnhum.2024.1469468 39980907 PMC11841423

[B22] Gupta U. Gupta B. (2016). Gender differences in psychophysiological responses to music listening. Music Med. 8, 53–64. 10.47513/mmd.v8i1.471

[B23] Hanser W. E. ter Bogt T. F. Van den Tol A. J. Mark R. E. Vingerhoets A. J. (2016). Consolation through music: a survey study. Musicae Sci. 20, 122–137. 10.1177/1029864915620264

[B24] Hu W. Zhang Z. Zhao H. Zhang L. Li L. Huang G.. (2023). EEG microstate correlates of emotion dynamics and stimulation content during video watching. Cerebral Cortex 33, 523–542. 10.1093/cercor/bhac082 35262653

[B25] Janata P. (2009). The neural architecture of music-evoked autobiographical memories. Cerebral Cortex 19, 2579–2594. 10.1093/cercor/bhp008 19240137 PMC2758676

[B26] Jäncke L. Kühnis J. Rogenmoser L. Elmer S. (2015). Time course of EEG oscillations during repeated listening of a well-known aria. Front. Hum. Neurosci. 9:401. 10.3389/fnhum.2015.00401 26257624 PMC4507057

[B27] Jiang H. Zhao S. Wu Q. Cao Y. Zhou W. Gong Y.. (2024). Dragon boat exercise reshapes the temporal-spatial dynamics of the brain. PeerJ 12:e17623. 10.7717/peerj.17623 38952974 PMC11216202

[B28] Kar S. Ganguly T. Roy S. Goswami A. (2015). Effect of indian classical music (raga therapy) on fentanyl, vecuronium, propofol requirements and cortisol levels in cardiopulmonary bypass. J. Anesth. Crit. Care Open Access 2:00047. 10.15406/jaccoa.2015.02.00047

[B29] Khanna A. Pascual-Leone A. Michel C. M. Farzan F. (2015). Microstates in resting-state EEG: current status and future directions. Neurosci. Biobehav. Rev. 49, 105–113. 10.1016/j.neubiorev.2014.12.010 25526823 PMC4305485

[B30] Kim K. Duc N. Choi M. Lee B. (2021). EEG microstate features according to performance on a mental arithmetic task. Sci. Rep. 11, 1–14. 10.1038/s41598-020-79423-7 33431963 PMC7801706

[B31] Koenig T. Lehmann D. Merlo M. C. Kochi K. Hell D. Koukkou M. (1999). A deviant EEG brain microstate in acute, neuroleptic-naive schizophrenics at rest. Eur. Arch. Psychiatry Clin. Neurosci. 249, 205–211. 10.1007/s004060050088 10449596

[B32] Koenig T. Prichep L. Lehmann D. Sosa P. V. Braeker E. Kleinlogel H.. (2002). Millisecond by millisecond, year by year: normative EEG microstates and developmental stages. Neuroimage 16, 41–48. 10.1006/nimg.2002.1070 11969316

[B33] Kucyi A. Salomons T. V. Davis K. D. (2013). Mind wandering away from pain dynamically engages antinociceptive and default mode brain networks. Proc. Nat. Acad. Sci. 110, 18692–18697. 10.1073/pnas.1312902110 24167282 PMC3832014

[B34] Liu H. Tang H. Wei W. Wang G. Du Y. Ruan J. (2021). Altered peri-seizure EEG microstate dynamics in patients with absence epilepsy. Seizure 88, 15–21. 10.1016/j.seizure.2021.03.020 33799135

[B35] Mammarella N. Fairfield B. Cornoldi C. (2007). Does music enhance cognitive performance in healthy older adults? the vivaldi effect. Aging Clin. Exp. Res. 19, 394–399. 10.1007/BF03324720 18007118

[B36] Markovic A. Kühnis J. Jäncke L. (2017). Task context influences brain activation during music listening. Front. Hum. Neurosci. 11:342. 10.3389/fnhum.2017.00342 28706480 PMC5489556

[B37] Mason M. F. Norton M. I. Van Horn J. D. Wegner D. M. Grafton S. T. Macrae C. N. (2007). Wandering minds: the default network and stimulus-independent thought. Science 315, 393–395. 10.1126/science.1131295 17234951 PMC1821121

[B38] Michel C. M. Koenig T. (2018). EEG microstates as a tool for studying the temporal dynamics of whole-brain neuronal networks: a review. Neuroimage 180, 577–593. 10.1016/j.neuroimage.2017.11.062 29196270

[B39] Milz P. Pascual-Marqui R. D. Achermann P. Kochi K. Faber P. L. (2017). The EEG microstate topography is predominantly determined by intracortical sources in the alpha band. Neuroimage 162, 353–361. 10.1016/j.neuroimage.2017.08.058 28847493

[B40] Murray M. M. Brunet D. Michel C. M. (2008). Topographic erp analyses: a step-by-step tutorial review. Brain Topogr. 20, 249–264. 10.1007/s10548-008-0054-5 18347966

[B41] Neubauer A. C. Fink A. (2009). Intelligence and neural efficiency. Neuroscience &Biobehav. Rev. 33, 1004–1023. 10.1016/j.neubiorev.2009.04.001 19580915

[B42] Nishida K. Morishima Y. Yoshimura M. Isotani T. Irisawa S. Jann K.. (2013). EEG microstates associated with salience and frontoparietal networks in frontotemporal dementia, schizophrenia and Alzheimer's disease. Clin. Neurophysiol. 124, 1106–1114. 10.1016/j.clinph.2013.01.005 23403263

[B43] Pal A. Behari M. Goyal V. Sharma R. (2021). Study of EEG microstates in Parkinson's disease: a potential biomarker? Cogn. Neurodyn. 15, 463–471. 10.1007/s11571-020-09643-0 34040672 PMC8131463

[B44] Pascual-Marqui R. D. Lehmann D. Faber P. Milz P. Kochi K. Yoshimura M.. (2014). The resting microstate networks (RMN): cortical distributions, dynamics, and frequency specific information flow. arXiv preprint arXiv:1411.1949.

[B45] Poulsen A. T. Pedroni A. Langer N. Hansen L. K. (2018). Microstate EEGlab toolbox: an introductory guide. BioRxiv, 289850. 10.1101/289850

[B46] Prete G. Croce P. Zappasodi F. Tommasi L. Capotosto P. (2022). Exploring brain activity for positive and negative emotions by means of EEG microstates. Sci. Rep. 12:3404. 10.1038/s41598-022-07403-0 35233057 PMC8888606

[B47] Putkinen V. Makkonen T. Eerola T. (2017). Music-induced positive mood broadens the scope of auditory attention. Soc. Cogn. Affect. Neurosci. 12, 1159–1168. 10.1093/scan/nsx038 28460035 PMC5490675

[B48] Rauscher F. H. Shaw G. L. Ky C. N. (1993). Music and spatial task performance. Nature 365, 611–611. 10.1038/365611a0 8413624

[B49] Rauscher F. H. Shaw G. L. Ky K. N. (1995). Listening to mozart enhances spatial-temporal reasoning: towards a neurophysiological basis. Neurosci. Lett. 185, 44–47. 10.1016/0304-3940(94)11221-4 7731551

[B50] Rideout B. E. Laubach C. M. (1996). EEG correlates of enhanced spatial performance following exposure to music. Percept. Mot. Skills 82, 427–432. 10.2466/pms.1996.82.2.427 8724912

[B51] Sachs M. E. Damasio A. Habibi A. (2015). The pleasures of sad music: a systematic review. Front. Hum. Neurosci. 9:404. 10.3389/fnhum.2015.00404 26257625 PMC4513245

[B52] Särkämö T. (2018). Cognitive, emotional, and neural benefits of musical leisure activities in aging and neurological rehabilitation: a critical review. Ann. Phys. Rehabil. Med. 61, 414–418. 10.1016/j.rehab.2017.03.006 28461128

[B53] Schellenberg E. G. Hallam S. (2005). Music listening and cognitive abilities in 10-and 11-year-olds: the blur effect. Ann. N. Y. Acad. Sci. 1060, 202–209. 10.1196/annals.1360.013 16597767

[B54] Schellenberg E. G. Nakata T. Hunter P. G. Tamoto S. (2007). Exposure to music and cognitive performance. Psychol. Music 35, 5–19. 10.1177/0305735607068885

[B55] Schiller B. Kleinert T. Teige-Mocigemba S. Klauer K. C. Heinrichs M. (2020). Temporal dynamics of resting EEG networks are associated with prosociality. Sci. Rep. 10:13066. 10.1038/s41598-020-69999-5 32747655 PMC7400630

[B56] Seitzman B. A. Abell M. Bartley S. C. Erickson M. A. Bolbecker A. R. Hetrick W. P. (2017). Cognitive manipulation of brain electric microstates. Neuroimage 146, 533–543. 10.1016/j.neuroimage.2016.10.002 27742598 PMC5321823

[B57] Shen X. Hu X. Liu S. Song S. Zhang D. (2020). “Exploring EEG microstates for affective computing: decoding valence and arousal experiences during video watching,” in 2020 42nd Annual International Conference of the IEEE Engineering in Medicine &Biology Society (EMBC) (IEEE), 841–846. 10.1109/EMBC44109.2020.9175482 33018116

[B58] Siritunga S. Wijewardena K. Ekanayaka R. Mudunkotuwa P. (2013). Effect of music on blood pressure, pulse rate and respiratory rate of asymptomatic individuals: a randomized controlled trial. Health 5, 59–64. 10.4236/health.2013.54A008

[B59] Skrandies W. (1990). Global field power and topographic similarity. Brain Topogr. 3, 137–141. 10.1007/BF01128870 2094301

[B60] Soni S. Muthukrishnan S. P. Samanchi R. Sood M. Kaur S. Sharma R. (2019). Pre-trial and pre-response EEG microstates in schizophrenia: an endophenotypic marker. Behav. Brain Res. 371:111964. 10.1016/j.bbr.2019.111964 31129232

[B61] Tait L. Tamagnini F. Stothart G. Barvas E. Monaldini C. Frusciante R.. (2020). EEG microstate complexity for aiding early diagnosis of Alzheimer's disease. Sci. Rep. 10, 1–10. 10.1038/s41598-020-74790-7 33077823 PMC7572485

[B62] Tarailis P. Koenig T. Michel C. M. Griškova-Bulanova I. (2023). The functional aspects of resting EEG microstates: a systematic review. Brain Topogr. 37, 181–217. 10.1007/s10548-023-00958-9 37162601

[B63] Taruffi L. Koelsch S. (2014). The paradox of music-evoked sadness: an online survey. PLoS ONE 9:e110490. 10.1371/journal.pone.0110490 25330315 PMC4203803

[B64] Taruffi L. Pehrs C. Skouras S. Koelsch S. (2017). Effects of sad and happy music on mind-wandering and the default mode network. Sci. Rep. 7:14396. 10.1038/s41598-017-14849-0 29089542 PMC5663956

[B65] Ter Bogt T. F. Vieno A. Doornwaard S. M. Pastore M. Van den Eijnden R. J. (2017). “You're not alone”: music as a source of consolation among adolescents and young adults. Psychol. Music 45, 155–171. 10.1177/0305735616650029

[B66] Terpou B. A. Shaw S. B. Théberge J. Férat V. Michel C. M. McKinnon M. C.. (2022). Spectral decomposition of EEG microstates in post-traumatic stress disorder. NeuroImage: Clin. 35:103135. 10.1016/j.nicl.2022.103135 36002969 PMC9421541

[B67] Trost W. Ethofer T. Zentner M. Vuilleumier P. (2012). Mapping aesthetic musical emotions in the brain. Cerebral Cortex 22, 2769–2783. 10.1093/cercor/bhr353 22178712 PMC3491764

[B68] Van den Tol A. J. Edwards J. (2013). Exploring a rationale for choosing to listen to sad music when feeling sad. Psychol. Music 41, 440–465. 10.1177/0305735611430433

[B69] Van den Tol A. J. Edwards J. Heflick N. A. (2016). Sad music as a means for acceptance-based coping. Musicae Scient. 20, 68–83. 10.1177/1029864915627844

[B70] Verrusio W. Ettorre E. Vicenzini E. Vanacore N. Cacciafesta M. Mecarelli O. (2015). The mozart effect: a quantitative EEG study. Conscious. Cogn. 35, 150–155. 10.1016/j.concog.2015.05.005 26036835

[B71] von Wegner F. Bauer S. Rosenow F. Triesch J. Laufs H. (2021). EEG microstate periodicity explained by rotating phase patterns of resting-state alpha oscillations. Neuroimage 224:117372. 10.1016/j.neuroimage.2020.117372 32979526

[B72] Wang L. Ding X. Zhang W. Yang S. (2021). Differences in EEG microstate induced by gaming: a comparison between the gaming disorder individual, recreational game users and healthy controls. IEEE Access 9, 32549–32558. 10.1109/ACCESS.2021.3060112

[B73] Whittle S. Yücel M. Yap M. B. Allen N. B. (2011). Sex differences in the neural correlates of emotion: evidence from neuroimaging. Biol. Psychol. 87, 319–333. 10.1016/j.biopsycho.2011.05.003 21600956

[B74] Wilkins R. W. Hodges D. A. Laurienti P. J. Steen M. Burdette J. H. (2014). Network science and the effects of music preference on functional brain connectivity: from beethoven to eminem. Sci. Rep. 4, 1–8. 10.1038/srep06130 25167363 PMC5385828

[B75] Wilson T. L. Brown T. L. (1997). Reexamination of the effect of mozart's music on spatial-task performance. J. Psychol. 131, 365–370. 10.1080/00223989709603522

[B76] Yeshurun Y. Nguyen M. Hasson U. (2021). The default mode network: where the idiosyncratic self meets the shared social world. Nat. Rev. Neurosci. 22, 181–192. 10.1038/s41583-020-00420-w 33483717 PMC7959111

[B77] Zanesco A. P. Skwara A. C. King B. G. Powers C. Wineberg K. Saron C. D. (2021). Meditation training modulates brain electric microstates and felt states of awareness. Hum. Brain Mapp. 42, 3228–3252. 10.1002/hbm.25430 33783922 PMC8193519

[B78] Zhang K. Shi W. Wang C. Li Y. Liu Z. Liu T.. (2021). Reliability of EEG microstate analysis at different electrode densities during propofol-induced transitions of brain states. Neuroimage 231:117861. 10.1016/j.neuroimage.2021.117861 33592245

[B79] Zulliger J. Diaz Hernandez L. Koenig T. (2022). Within and between subject spectral fingerprints of EEG-microstate parameters. Brain Topogr. 35, 277–281. 10.1007/s10548-022-00896-y 35414139 PMC9098597

