# Supplementary material for: EEG microstates dynamics of happiness and sadness during music listening
Source: Front Hum Neurosci. 2025 Jun 18;19:1472689. doi: 10.3389/fnhum.2025.1472689 (PMC12213508; doi:10.3389/fnhum.2025.1472689)
Supplement: Supplementary file 1 [file Data_Sheet_1.PDF]

Supplementary Information:

Examining EEG Microstates Dynamics of Happiness and Sadness during Music Listening

Ashish gupta[1], Chandan Kumar Srivastava[2], Braj Bhushan[3], and Laxmidhar Behera[1,4*]

[1]Department of Electrical Engineering, Indian Institute of Technology, Kanpur, India.

[2]Department of Humanities and Social Sciences, Indian Institute of Technology, Bombay, India.

[3]Department of Humanities and Social Sciences, Indian Institute of Technology, Kanpur, India.

[4]School of Computing and Electrical Engineering, Indian Institute of Technology, Mandi, India.

***Correspondence and requests for materials should be addressed to L.D. (lbehera@iitk.ac).**

1. Class D microstate's parameter as function of time-period during Raga darbari listening (happy music).

Fig S1:

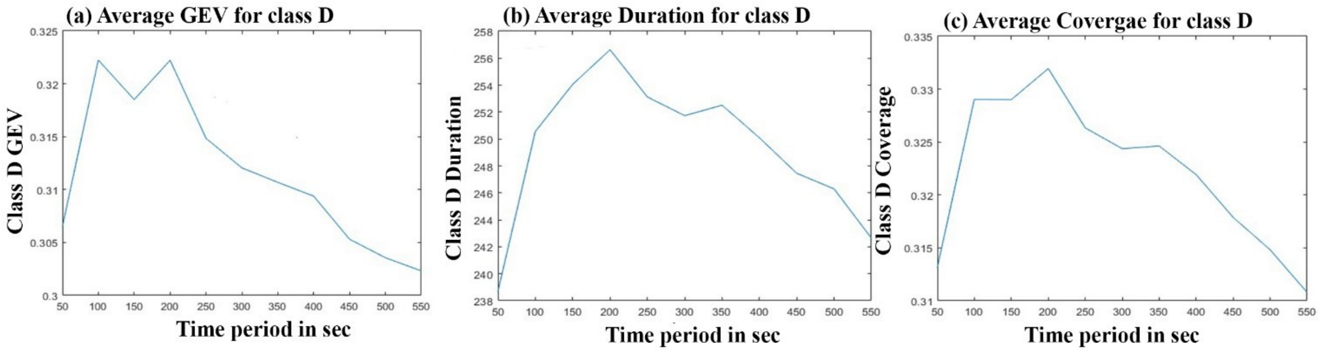

Fig S1(a-c) shows the GEV, duration, and coverage of class D microstate during happy music listening as function of time-period.

Class D microstate is linked to attention. Results signify that attention reaches maximum at 200 sec time-period and thereafter it decreases during happy music listening. Thus, for comparison with the baseline resting state (BL) and post music silence condition (PMS), we selected first 200 sec of music for analysis. 200 sec of baseline and 200 sec post music silence condition were further selected for the analysis.

Since the effect of music is short term, post music listening, we selected 200 sec post music silence condition. Accordingly, 200 sec for BL and Mus conditions as discussed above.

To keep the uniformity between experiment 1 and 2, we selected first 200 sec of sad music listening for comparative analysis with baseline and post music silence, like as we did for happy music listening analysis.

2. The topological orientation of the Microstate maps:

Koenig et. al.[i] classified four microstate maps of the brain into classes A, B, C, and D according to their topological orientation (Fig S2). Specifically,
microstate map A shows a left-right orientation,
microstate map B displays a right-left orientation,
microstate map C demonstrates an anterior-posterior orientation, and
microstate map D reveals a fronto-central maximum.
Following studies have consistently adhered to this labelling convention[ii].
We categorized the acquired microstates in our study as classes A, B, C, and D based on their topographical orientation, as outlined by Koenig et. al.[i,iii] in line with earlier studies[iv,v,vi](Fig S2-S4).

Fig S2: Four microstate maps as per the study by Koeing et. al.[i].

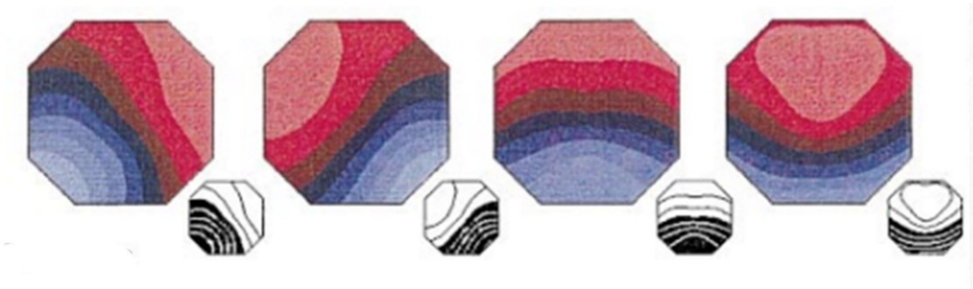

C lass A Class B Class C Class D

Fig S3: Four microstate maps as per a recent study on the DEAP datasets[iv].

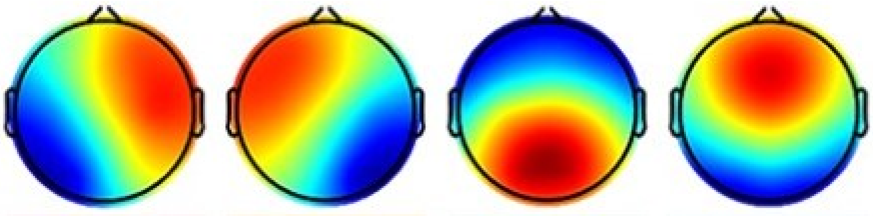

C lass A Class B Class C Class D

Fig S4: Visual representation of the topography of the four canonical microstate maps, identified in numerous independent studies with varying numbers of electrodes, participants, and filter settings[ii]. Despite their distinctiveness, these four microstate maps exhibit a high level of reproducibility across different studies.

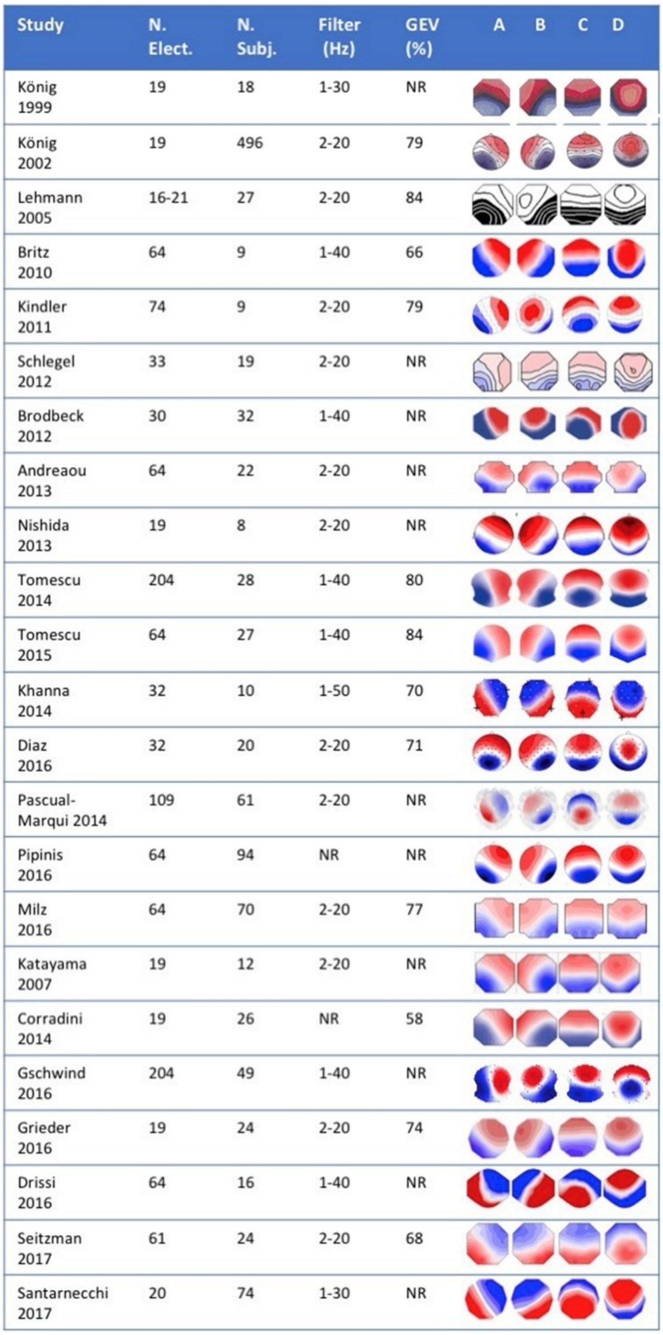

Study	N. Elect.	N. Subj.	Filter (Hz)	GEV (%)	A	B	C	D
König 1999	19	18	1-30	NR				
König 2002	19	496	2-20	79				
Lehmann 2005	16-21	27	2-20	84				
Britz 2010	64	9	1-40	66				
Kindler 2011	74	9	2-20	79				
Schlegel 2012	33	19	2-20	NR				
Brodbeck 2012	30	32	1-40	NR				
Andreaou 2013	64	22	2-20	NR				
Nishida 2013	19	8	2-20	NR				
Tomescu 2014	204	28	1-40	80				
Tomescu 2015	64	27	1-40	84				
Khanna 2014	32	10	1-50	70				
Diaz 2016	32	20	2-20	71				
Pascual-Marqui 2014	109	61	2-20	NR				
Pipinis 2016	64	94	NR	NR				
Milz 2016	64	70	2-20	77				
Katayama 2007	19	12	2-20	NR				
Corradini 2014	19	26	NR	58				
Gschwind 2016	204	49	1-40	NR				
Grieder 2016	19	24	2-20	74				
Drissi 2016	64	16	1-40	NR				
Seitzman 2017	61	24	2-20	68				
Santarnecchi 2017	20	74	1-30	NR				

Four microstates' maps obtained in the current study is in line with four microstate maps obtained in earlier studies[i,ii,iii,iv,v,vi]. Thus, validating the microstates obtained in the current study.

[i] Koenig, T., Lehmann, D., Merlo, M. C., Kochi, K., Hell, D., & Koukkou, M. (1999). A deviant EEG brain microstate in acute, neuroleptic-naive schizophrenics at rest. *European archives of psychiatry and clinical neuroscience*, *249*, 205-211.

[ii] Michel, C. M., & Koenig, T. (2018). EEG microstates as a tool for studying the temporal dynamics of whole-brain neuronal networks: a review. *Neuroimage*, *180*, 577-593.

[iii] Koenig, T., Prichep, L., Lehmann, D., Sosa, P. V., Braeker, E., Kleinlogel, H., ... & John, E. R. (2002). Millisecond by millisecond, year by year: normative EEG microstates and developmental stages. *Neuroimage*, *16*(1), 41-48.

[iv] Hu, W., Zhang, Z., Zhao, H., Zhang, L., Li, L., Huang, G., & Liang, Z. (2023). EEG microstate correlates of emotion dynamics and stimulation content during video watching. *Cerebral Cortex*, *33*(3), 523-542.

[v] Liu, H., Tang, H., Wei, W., Wang, G., Du, Y., & Ruan, J. (2021). Altered peri-seizure EEG microstate dynamics in patients with absence epilepsy. *Seizure*, *88*, 15-21.

[vi] Pal, A., Behari, M., Goyal, V., & Sharma, R. (2021). Study of EEG microstates in Parkinson's disease: a potential biomarker?. *Cognitive Neurodynamics*, *15*, 463-471.
